# Local Transcriptional Control of YUCCA Regulates Auxin Promoted Root-Growth Inhibition in Response to Aluminium Stress in *Arabidopsis*

**DOI:** 10.1371/journal.pgen.1006360

**Published:** 2016-10-07

**Authors:** Guangchao Liu, Shan Gao, Huiyu Tian, Wenwen Wu, Hélène S. Robert, Zhaojun Ding

**Affiliations:** 1 The Key Laboratory of Plant Cell Engineering and Germplasm Innovation, Ministry of Education, College of Life Science, Shandong University, Jinan, People’s Republic of China; 2 Mendel Centre for Genomics and Proteomics of Plants Systems, CEITEC MU—Central European Institute of Technology, Masaryk University, Brno, Czech Republic; National University of Singapore and Temasek Life Sciences Laboratory, SINGAPORE

## Abstract

Auxin is necessary for the inhibition of root growth induced by aluminium (Al) stress, however the molecular mechanism controlling this is largely unknown. Here, we report that YUCCA (YUC), which encodes flavin monooxygenase-like proteins, regulates local auxin biosynthesis in the root apex transition zone (TZ) in response to Al stress. Al stress up-regulates *YUC3/5/7/8/9* in the root-apex TZ, which we show results in the accumulation of auxin in the root-apex TZ and root-growth inhibition during the Al stress response. These Al-dependent changes in the regulation of *YUC*s in the root-apex TZ and YUC-regulated root growth inhibition are dependent on ethylene signalling. Increasing or disruption of ethylene signalling caused either enhanced or reduced up-regulation, respectively, of *YUC*s in root-apex TZ in response to Al stress. In addition, ethylene enhanced root growth inhibition under Al stress was strongly alleviated in *yuc* mutants or by co-treatment with yucasin, an inhibitor of YUC activity, suggesting a downstream role of YUCs in this process. Moreover, ethylene-insensitive 3 (EIN3) is involved into the direct regulation of *YUC9* transcription in this process. Furthermore, we demonstrated that PHYTOCHROME INTERACTING FACTOR4 (PIF4) functions as a transcriptional activator for *YUC5*/*8*/*9*. PIF4 promotes Al-inhibited primary root growth by regulating the local expression of *YUCs* and auxin signal in the root-apex TZ. The Al–induced expression of PIF4 in root TZ acts downstream of ethylene signalling. Taken together, our results highlight a regulatory cascade for YUCs-regulated local auxin biosynthesis in the root-apex TZ mediating root growth inhibition in response to Al stress.

## Author Summary

The phytohormone auxin, which is synthesized mainly through TRYPTOPHAN AMINOTRANSFERASE OF ARABIDOPSIS 1 (TAA1) and YUCCA (YUC) flavin monooxygenase-like proteins, has an important role in the inhibition of root growth induced by aluminium (Al) stress. TAA1 was recently shown to be locally induced in the root-apex transition-zone thus involves in aluminium-induced *Arabidopsis* root growth inhibition. Here, we report that YUCCA (YUC) regulates local auxin biosynthesis in the root apex transition zone (TZ) and controls root growth in response to Al stress. Furthermore, EIN3 and PIF4 were found to transcriptionally regulate Al-induced *YUCCA* expression and thus involve in Al-induced auxin accumulation in root TZ and root growth inhibition in *Arabidopsis thaliana*

## Introduction

Aluminium is highly abundant in the soil, but only presents toxicity problems to plants in acid (pH≤5) soils, where it becomes solubilized into the Al^3+^ ion. Relatively modest levels of Al^3+^ in the soil are sufficient to inhibit root growth in most species [1,2,3,4]. As a result, gaining an understanding of how Al compromises root growth has preoccupied a substantial body of crop research. The root tip is recognized as the major target of Al stress [5,6,7]; in maize, common bean and *Arabidopsis thaliana*, the distal portion of the root-apex transition zone (TZ), located between the apical meristem and the basal elongation region, has been shown to be the most sensitive part of the root [7,8,9,10,11,12]. The same part of the root in sorghum is the site for reactive oxygen species production, a class of molecules, which cause root growth inhibition under Al stress [13].

A variety of plant growth and developmental processes are mediated by the phytohormone auxin, which is present for the most part as indole-3-acetic acid (IAA). IAA is mainly synthesized via a tryptophan-dependent pathway [14]. The YUCCA (YUC) family of flavin-containing mono-oxygenases and the TRYPTOPHAN AMINOTRANSFERASE OF ARABIDOPSIS (TAA) family of aminotransferases are both key enzymes in this pathway [15,16,17,18,19]. The *Arabidopsis* genome harbours three *TAA* genes, and eleven *YUC* genes. TAA enzymes catalyse the conversion of tryptophan to indole-3-pyruvate (IPyA), while YUCs are involved in the conversion of IPyA to IAA, a rate-limiting step in the IPyA pathway [18,19,20,21,22].

Local auxin biosynthesis mediated tissue or cellular auxin responses control many plant growth and developmental responses [15,17,18,19,23]. During plant embryogenesis, YUC1, YUC3, YUC4, YUC8 and YUC9 were found to be involved in the control of localized auxin biosynthesis in early embryos [24]. Local auxin biosynthesis mediated plant growth and development is also regulated by tissue or cellular specific transcription factors. For example, AP2 PLETORA transcription factors have been implicated as regulators of lateral organ out-growth through the regulation of localized auxin synthesis controlled by YUCs [25]. The basic helix–loop–helix transcription factors, phytochrome-interacting factor 4 (PIF4) and PIF5, through the regulation of *TAA1* and *YUC8*, integrates temperature into the auxin signaling pathway to control *Arabidopsis* hypocotyl growth [26,27,28,29].

Phytohormones, particularly auxin and ethylene, play critical roles in modulating root growth in response to Al stress. Previous study indicated that ethylene enhanced Al induced inhibition of root elongation, and exogenous application of aminoethoxyvinylglycine (AVG) and Co^2+^, ethylene biosynthesis inhibitors, or ethylene signaling mutants such as *ein2* and *ein3-1 eil1-1* mutants markedly alleviates the Al induced inhibition of root elongation [7,30,31]. Auxin was also found to play a positive role in the Al-induced root growth inhibition [7,32]. Consistently, blocking auxin signaling with the auxin antagonist a-(phenylethyl-2-one)-indole-3-acetic acid (PEO-IAA), a molecule that blocks the auxin binding sites of TIR1/AFB auxin receptors [33,34], or in auxin related mutants such as *slr-1* and *arf10 arf16* greatly enhances root growth inhibition in response to Al stress [7]. TAA1-mediated localized auxin synthesis has been invoked to explain the basis of both shade avoidance and Al-induced root growth inhibition [7,19]. Exposure of *Arabidopsis* roots to Al stress has been shown to enhance auxin signalling in the root-apex TZ, a process which is dependent on TAA1-regulated auxin synthesis [7]. This study explores the roles of YUCs (YUC3, YUC5, YUC7, YUC8 and YUC9), which were recently shown to regulate root development [35], in the Al stress-induced inhibition of root growth. The results show that YUCCA controls Al-inhibited primary root growth through the regulation of local auxin biosynthesis in the root-apex TZ in *Arabidopsis thaliana*. Ethylene-insensitive3 (EIN3) [36] and PHYTOCHROME INTERACTING FACTOR4 (PIF4) [29] functions as a transcriptional activator for *YUC5*/*8*/*9* in this process.

## Results

### YUCs are involved in Al stress-induced root growth inhibition

**Fig 1:**
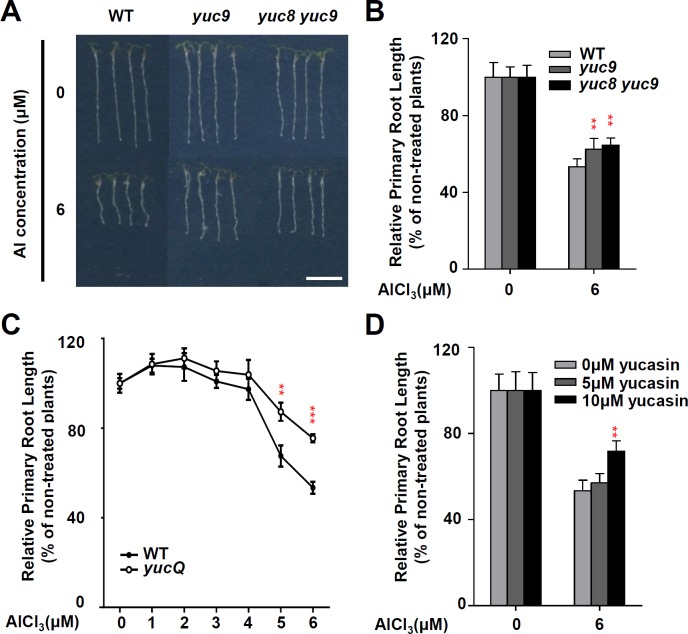
YUC regulates Al stress regulated root growth inhibition. **(A, B)** Root growth of WT and *yuc* mutant seedlings after a seven-day exposure to 0 or 6 μM AlCl_3_. Three independent experiments were done, each with three replicates. Plants were grown at 22°C in long-day growth conditions. Bar = 1 cm. **(C)** Root growth of WT (*DR5rev*:*GFP*) and *yucQ/DR5rev*:*GFP* plants after a seven-day exposure of 0 to 6 μM AlCl_3_. (D) Effect on the Al-induced inhibition of root growth of WT by addition the YUC inhibitor yucasin. Root growth measured after a seven-day exposure to 0 or 6 μM AlCl_3_ in the presence of 0 to 10 μM yucasin. Three independent experiments were done, each with three replicates. Error bars represent Student’s *t* test confidence intervals (n = 9). Statistical difference from expected indicated by asterisks (Fisher’s exact test, **P < 0.01, ***P < 0.005).

To understand the roles of YUCs, which regulate the rate-limiting step of auxin biosynthesis via the conversion of IPyA to IAA, in Al stress-regulated root growth inhibition, we examined the phenotypes of *YUC* mutants such as *yuc3*, *yuc7*, *yuc8*, *yuc9*, *yuc8 yuc9* and *yucQ (yuc3/5/7/8/9)* under Al stress treatment. Al-dependent root growth inhibition was least severe in *yuc9* and *yuc8 yuc9* double mutants, while its extent in the other four single mutants was similar to that of the wild type (WT) control (Fig 1A and 1B, S1 Fig). Given the functional redundancy among these five YUC proteins, attention was focussed on the multiple *yucQ* mutant, in which all five *YUC* genes have been silenced [35]. Consistent with the previous study which showed the defective root phenotypes [35], we also observed the short root length phenotype in *yucQ* in the presence of sucrose. However, in the absence of sucrose which was used for the Al-treatment, we didn’t see the clear root length phenotype (S2 Fig). When the roots were exposed to levels of Al >1μM, *yucQ* is defective in Al-induced growth inhibition (Fig 1C). The more pronounced phenotype in *yucQ* demonstrated the redundant action of YUC in Al stress-regulated root growth inhibition. When yucasin (5-(4-chlorophenyl)-4H-1,2,4-triazole-3-thiol), an inhibitor of YUC activity [37], was introduced in the growth medium, the effect of Al stress on root-growth inhibition was strongly reduced (Fig 1D). These data indicate that the YUCs-dependent auxin production forms part of the cellular machinery responsible for Al stress-induced root growth inhibition.

### YUCs regulate Al-induced local auxin response in the root-apex TZ

**Fig 2:**
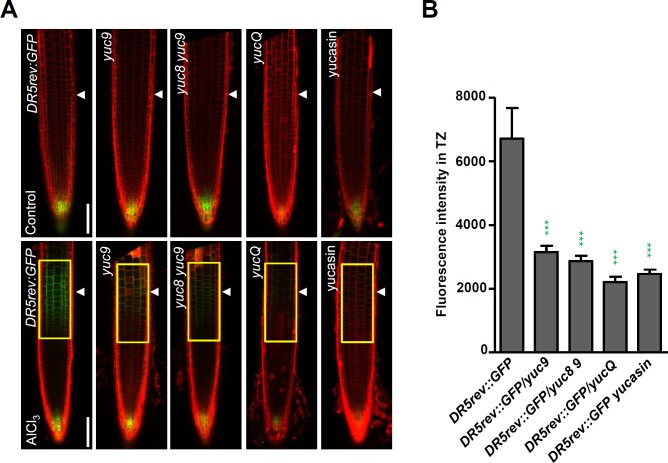
YUCs regulate Al induced local auxin response in root TZ. **(A)** Five-day old *DR5rev*:*GFP*, *DR5rev*:*GFP/yuc9*, *DR5rev*:*GFP/yuc8 yuc9* and *DR5rev*:*GFP/yucQ* seedlings were exposed or not (control) to 25 μM AlCl_3_ for two hours. Four-day old transgenic *DR5rev*:*GFP* seedlings were pre-treated with 10 μM yucasin for 1 day, then were co-treated with 25 μM AlCl_3_ for two hours or not (control). The upper row shows expression of *DR5rev*:*GFP*, *DR5rev*:*GFP/yuc9*, *DR5rev*:*GFP/yuc8 yuc9 and DR5rev*:*GFP/yucQ* controls while the lower row shows the expression of these transgenic seedlings exposed to 25 μM AlCl_3_. Cell boundaries appear red following propidium iodide staining. The root-apex TZ is marked by white arrowheads. Scale bar: 100 μm. **(B)** Quantification of the Al-induced fluorescence intensity in the TZ of *DR5rev*:*GFP*, *DR5rev*:*GFP/yuc9*, *DR5rev*:*GFP/yuc8 yuc9*, *DR5rev*:*GFP/yucQ* and 10 μM yucasin pre-treated *DR5rev*:*GFP* seedlings (around 30 seedlings were measured in each material). The detected fluorescence region in TZ is marked by yellow rectangles. Cell boundaries appear red following propidium iodide staining. The TZ is marked by white arrowheads. Statistical difference from detected fluorescence is indicated by asterisks (Fisher’s exact test, ***P < 0.001).

To test if YUC-regulated auxin biosynthesis is involved in the local auxin response in the root-apex TZ, and thus in root growth inhibition in response to Al stress, we first examined the expression of the auxin responsive reporter *DR5rev*:*GFP* in *yuc9*, *yuc8 yuc9*, *yucQ* mutants (Fig 2). The results show that the strong Al stress-induced DR5rev:GFP signals produced in the root-apex TZ of a AlCl_3_ treated-WT root was remarkably attenuated in *yuc9*, *yuc8 yuc9* and *yucQ* mutant backgrounds (Fig 2). The presence of yucasin ensured that YUC activity was effectively compromised [37], thereby alleviating the Al stress-induced inhibition of root growth (Fig 1D); it also strongly reduced the level of Al treatment–induced *DR5* expression in the WT root-apex TZ (Fig 2). Thus this indicates that YUC activity is involved in the auxin response in the root-apex TZ when plants are subjected to Al stress.

### *YUC*s are locally induced in the root-apex TZ in presence of Al stress

**Fig 3:**
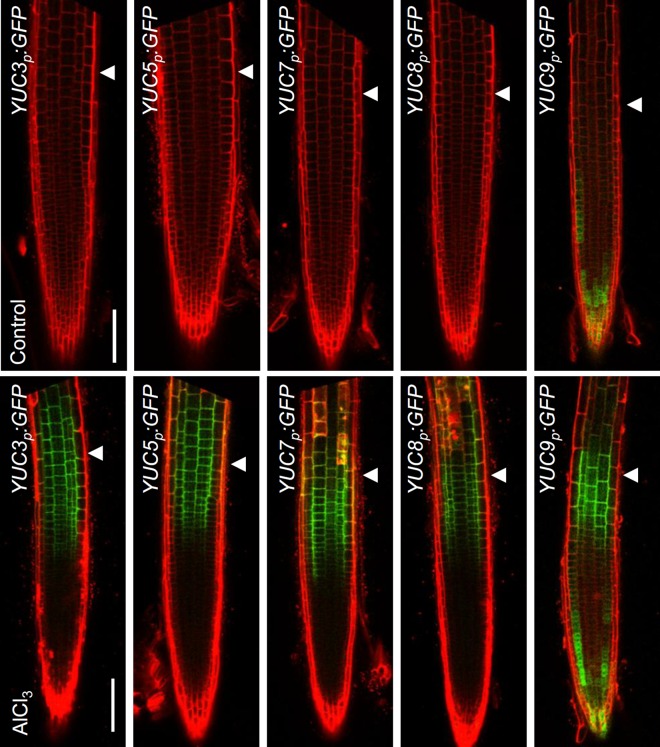
Al stress up-regulated the expression of *YUC*s in the root-apex TZ. The expression of the *YUCp*:*eGFP-GUS* transgenes in epidermis of the roots exposed to 25 μM AlCl_3_ for two hours (lower row). Controls are untreated roots (upper row). Cell boundaries appeared red following propidium iodide staining. The root-apex TZ is marked by white arrowheads. Scale bar: 100 μm.

To address how YUCs regulate the local auxin signaling in the root-apex TZ in the presence of Al stress, the effect of exposure to Al stress on the spatial expression of the *YUC* gene*s* was analysed by monitoring the expression of the *YUCp*:*eGFP-GUS* (*YUC3*, *YUC5*, *YUC7*, *YUC8* and *YUC9*) transgenes. Under non-stressed conditions, GFP signal was detected in the root tips of *YUC9p*:*eGFP-GUS* transgenic plants, but not initially in those of either *YUC3p*:*eGFP-GUS*, *YUC5p*:*eGFP-GUS*, *YUC7p*:*eGFP-GUS* or *YUC8p*:*eGFP-GUS*; however, after a 2 hours-exposure to Al stress, GFP signals did develop in the root-apex TZ of each of the transgenic lines (Fig 3 and S3 Fig). The GUS-staining assay showed the similar results when exposed to Al stress (S4 Fig). Furthermore, decreasing the pH of the medium from 5.5 to 4.2 did not affect the expression of *YUCp*:*eGFP-GUS* (*YUC3*, *YUC5*, *YUC7*, *YUC8* and *YUC9*) transgenes (S5 Fig), suggesting that exogenous Al, not protons, up-regulates *YUCs*, driving up the accumulation of auxin in the root-apex TZ and finally inhibiting root growth. In summary, similarly to what was demonstrated for TAA1 [7], the *YUC* genes are also specifically induced in the root-apex TZ in response to Al stress.

### The Al-regulated local expression of the *YUC* genes is ethylene dependent

**Fig 4:**
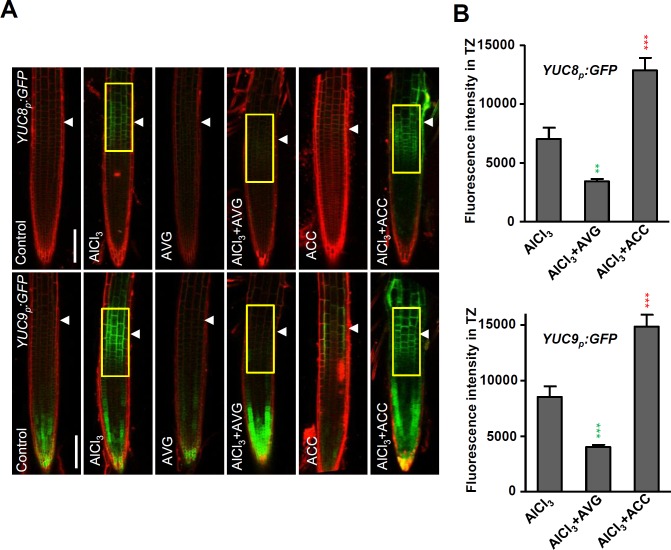
Al regulated local up-regulation of *YUC*s is ethylene dependent. (A) The expression of *YUCp*:*GFP-GUS* transgenes in the root-apex epidermis in presence of Al and either ACC or AVG. Three-day old transgenic *YUCp*:*GFP-GUS* seedlings were pre-treated with 1 μM AVG or 1 μM ACC for 1 day, then the seedlings were treated with 1 μM AVG or 1 μM ACC in the presence or not of 25 μM AlCl_3_ for 2 hours. Cell boundaries appear red following propidium iodide staining. The root- apex TZ is marked by white arrowheads. Scale bar: 100μm. **(B)** Quantification of the Al-induced fluorescence intensity in the TZ of *YUC8p*:*GFP-GUS* and *YUC8p*:*GFP-GUS* in (A). Around 30 seedlings were measured in each material. The detected fluorescence region in TZ is marked by yellow rectangles. Cell boundaries appear red following propidium iodide staining. Statistical difference from detected fluorescence is indicated by asterisks (Fisher’s exact test, ***P < 0.001).

Auxin acts downstream of ethylene to regulate Al-induced root growth inhibition has been reported [7]. A comparison of the Al stress-induced expression of the *YUCp*:*eGFP-GUS* (*YUC3*, *YUC5*, *YUC7*, *YUC8* and *YUC9*) transgenes in the presence of either 1-aminocyclopropane-1-carboxylic acid (ACC) (the precursor of ethylene synthesis) or AVG (an inhibitor of ethylene synthesis) showed that the induction of GFP signals by Al treatment in the root-apex TZ was intensified by the former, but repressed by the latter in every case (Fig 4 and S6 Fig). These results indicate that the Al stress-regulated up-regulation of the *YUC* genes in the root-apex TZ is modulated by an ethylene-dependent process.

### The Al-regulated local expression of *YUC9* is directly activated by EIN3

**Fig 5:**
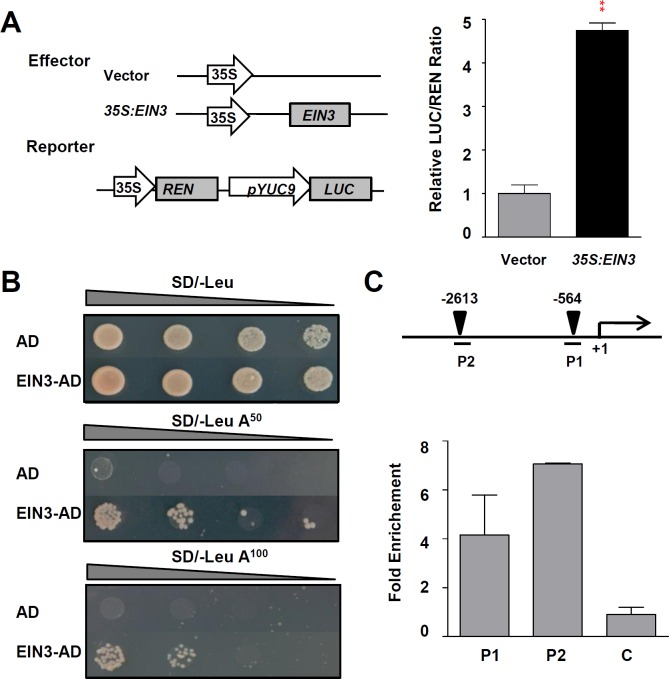
EIN3 is a transcriptional activator of *YUC9*. **(A)** Left panel: Schematic diagrams of effector and reporter constructs used in the transient dual-luciferase assays. *CaMV 35S* promoter driving *EIN3* (*35S*:*EIN3*) was used as effector, and empty vector as a negative control. Promoter fragments of *YUC9* were used to make the *YUC9p-LUC* reporter. Right panel: Transient dual-luciferase assay shows that *EIN3* transactivates the promoter of *YUC9* in *Arabidopsis* protoplasts. Data represent the means of three biological replicates. Error bars represent Student’s *t* test confidence intervals (n ≥ 9). Statistical significant difference indicated by asterisks (Fisher’s exact test, **P < 0.01). **(B)** Physical interactions of EIN3 with *YUC9* promoter in Y1H assays. Yeast expression plasmids pGADT7-EIN3 were reintroduced into yeast strain Y1H Gold carrying the reporter gene *AbAr* under the control of the *YUC9* promoter. The transformants were screened for their growth on the yeast synthetic defined media (SD/-Leu) in the presence of 50 or 100 ng ml^-1^ Aureobasidin A (AbA) for stringent selection. The empty vector pGADT7 was included as a negative control. Yeast cultures were diluted (1:10 successive dilution series) spotted onto plates. **(C)** EIN3 associated with the promoter of *YUC9* in ChIP-qPCR assay. Up panel: Schematic diagrams of *YUC9* promoter of showing the potential EIN3 binding site (black triangles). The translational start sites (ATG) are shown as +1. Numbers above the diagram indicated the distance away from ATG. DNA fragments (P1 and P2) were used for ChIP. Chromatins isolated from *35S*:*EIN3-GFP* transgenic line and *35S*:*GFP* control were immunoprecipitated with anti-GFP antibody followed by qPCR to amplify P1 and P2 regions. Segment C located in the coding region was used as negative control. Input sample was used to normalize the qPCR results in each ChIP. Fold enrichment was presented as a ratio of normalized results from *35S*:*EIN3-GFP* plants and *35S*:*GFP*. Data are mean ± SD.

Al-induced root growth inhibition was alleviated in the double mutant *ein3-1 eil1-1* as compared with the WT control, and inhibition of root growth in response to Al stress depends on ethylene signalling [7]. Thus, EIN3 and EIL1 may play an important role in the Al-regulated local up-regulation of *YUCs*. Therefore, we examined the expression patterns of *EIN3* and *EIL1* in response to Al stress using *EIN3p*:*GFP* and *EIL1p*:*GFP* transgenic lines. The results showed that Al stress induced a clear local up-regulation of *EIN3p*:*GFP* and *EIL1p*:*GFP* in the root-apex TZ (S7 Fig). EIN3/EIL1 transcription factors were reported to bind to a consensus DNA sequence of A[CT]G[AT]A[CT]CT [38,39]. Up to 3.0 Kb fragments upstream of the start codon (ATG) in the *YUC3*/*5*/*7*/*8*/*9* genes were analyzed with the Promomer tool database (http://bar.utoronto.ca/ntools/cgi-bin/BAR_Promomer.cgi) to identify potential EIN3 binding sites (EBS) on these promoters. Two putative EBS have been identified in the promoter of *YUC9* (S1 Material). Therefore, EIN3 may directly regulate *YUC9* expression through binding to its promoter. To test this possibility, we used transient dual-luciferase assays. By fusing *YUC9* promoter with Luciferase gene and testing its activation by overexpressing EIN3 in *Arabidopsis* protoplasts, we found that EIN3 could dramatically increase *YUC9* promoter activity (Fig 5A). To identify whether EIN3 has DNA binding activity to the *YUC9* promoter, we carried out yeast one-hybrid assay. Bait constructs containing promoter fragments of *YUC9* was prepared and the effector AD-EIN3 construct was generated. The results showed that EIN3 can physically bind to the *YUC9* promoter (Fig 5B). Consistently, chromatin immunoprecipitation–quantitative PCR (ChIP-qPCR) assay revealed the association of EIN3 protein with *YUC9* promoters in *35S*:*EIN3-GFP* transgenic *Arabidopsis* (Fig 5C). The strong Al stress-induced *YUC9*:*eGFP-GUS* signals produced in the root-apex TZ in Al treated-WT roots were remarkably attenuated in the *ein3-1 eil1-1* mutant backgrounds (S8 Fig). Therefore, EIN3 may actively regulate the Al-regulated local expression of the *YUC9* by directly binding to the *YUC9* promoter.

### YUCs-mediated local auxin biosynthesis and thus root growth inhibition in response to Al are ethylene-dependent

**Fig 6:**
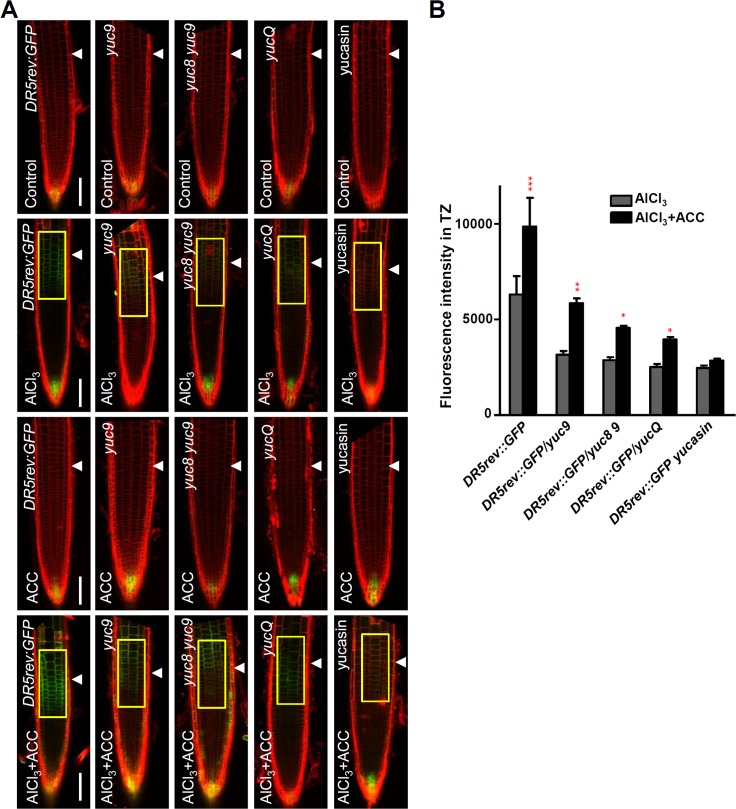
YUCs-dependent auxin biosynthesis contributes to ethylene enhanced local auxin signaling in root-apex TZ under Al stress. (A) The expression of *DR5rev*:*GFP*, *DR5rev*:*GFP/yuc9*, *DR5rev*:*GFP/yuc8 yuc9* and *DR5rev*:*GFP/yucQ* transgenes, or of *DR5rev*:*GFP* after yucasin treatment, in the epidermis of the root-apex in the presence of Al and/or ACC. Single treatments are performed on five-day old seedlings with 25 μM AlCl_3_ for 2 hours or on three-day old seedlings with 1 μM ACC or 10 μM yucasin for 2 hours. Co-treatments were done on three-day old transgenic seedlings (30 seedlings were analysed for each genotype/treatment combination) with pre-treatment with 1 μM ACC or 10 μM yucasin for 2 days, and co-treatment with 1 μM ACC or 10 μM yucasin and 25 μM AlCl_3_ for 2 hours. Cell boundaries appear red following propidium iodide staining. The root-apex TZ is marked by white arrowheads. Scale bar: 100μm. **(B)** Quantification of the Al-induced fluorescence intensity in the TZ of *DR5rev*:*GFP*, *DR5rev*:*GFP/yuc9*, *DR5rev*:*GFP/yuc8 yuc9* and *DR5rev*:*GFP/yucQ* transgenes, or of *DR5rev*:*GFP* after yucasin treatment in (A). Around 30 seedlings were measured in each material. The detected fluorescence region in TZ is marked by yellow rectangles. Cell boundaries appear red following propidium iodide staining. Statistical difference from detected fluorescence is indicated by asterisks (Fisher’s exact test, ***P < 0.001).

To address if YUCs-mediated local auxin biosynthesis and thus root growth inhibition in response to Al exposure acts downstream of ethylene signaling, the *DR5rev*:*GFP* expression in the WT and *yuc* mutants (*yuc9*, *yuc8 yuc9* and *yucQ*) root-apex TZ upon Al stress and in the presence of ACC was compared. The AlCl_3_/ACC co-treatment resulted in an intensification of the GFP signals throughout the root tip, but the level of enhancement was more modest in the mutant’s root-apex TZs. Furthermore, in the presence of yucasin, the ACC effect on auxin signalling was strongly attenuated (Fig 6). This result implies that the YUCs are involved in the ethylene positive modulation of auxin signalling in the root-apex TZ.

**Fig 7:**
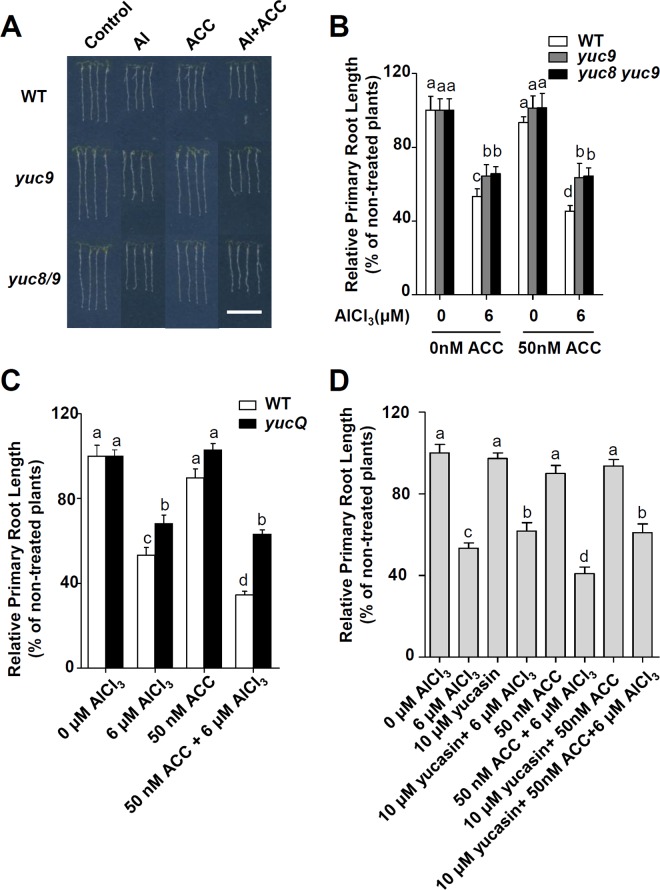
YUCs mediate ethylene signaling to enhance root growth inhibition in response to Al stress. **(A, B)** Root growth of WT (Col-0), *yuc9* and *yuc8 yuc9* seedlings after a seven-day exposure to 6 μM AlCl_3_, 50 nM ACC or co-treatment. Three independent experiments were done, each with three replicates. Plants were grown at 22°C in long days growth conditions. Bar = 1 cm. Error bars in **(B)** indicate mean ±SD (n≥30). Statistical significance was determined by two-way ANOVA with multiple comparison correction by Tukey HSD. Different letters indicate significance groups (P<0.05). **(C)** Root growth of WT (*DR5rev*:*GFP*), *DR5rev*:*GFP/yucQ* seedlings after a seven-day exposure to 6 μM AlCl_3_, 50 nM ACC or co-treatment. Three independent experiments were done, each with three replicates. Error bars indicate mean ±SD (n≥20). Statistical significance was determined by two-way ANOVA with multiple comparison correction by Tukey HSD. Different letters indicate significance groups (P<0.05). **(D)** Root growth of WT (Col-0) plants after a seven-day exposure to 6 μM AlCl_3_ in the presence or not of 10 μM yucasin and/or 50 nM ACC. Three independent experiments were done, each with three replicates. Bar = 1 cm. Error bars in indicate mean ±SD (n≥12). Statistical significance was determined by one-way ANOVA with multiple comparison correction by Tukey HSD. Different letters indicate significance groups (P<0.05).

The attenuated response of Al stressed *yuc* mutants after ACC treatment was monitored to determine whether YUCs-mediated auxin synthesis in the root-apex TZ (and thus root growth inhibition) acts downstream of ethylene signalling. The supply of a low concentration (50 nM) of ACC had no effect on the root growth of either WT or *yuc* mutant plants (Fig 7A and 7B), but Al co-treatment had a pronounced inhibitory effect on the WT root but not on the *yuc* mutant roots (Fig 7A–7C). Moreover the addition of 10 μM yucasin lifted the inhibition to root growth imposed on WT plants exposed to 50 nM ACC and 6 μM AlCl_3_. Note that this concentration of yucasin had no effect on the root growth of non-stressed WT plants (Fig 7D). We conclude that a low auxin level in root-apex TZ caused by a reduced auxin biosynthesis lead to a resistance to ACC-enhancement of Al stress-induced root growth.

### PIF4 is involved in Al-inhibited primary root growth

**Fig 8:**
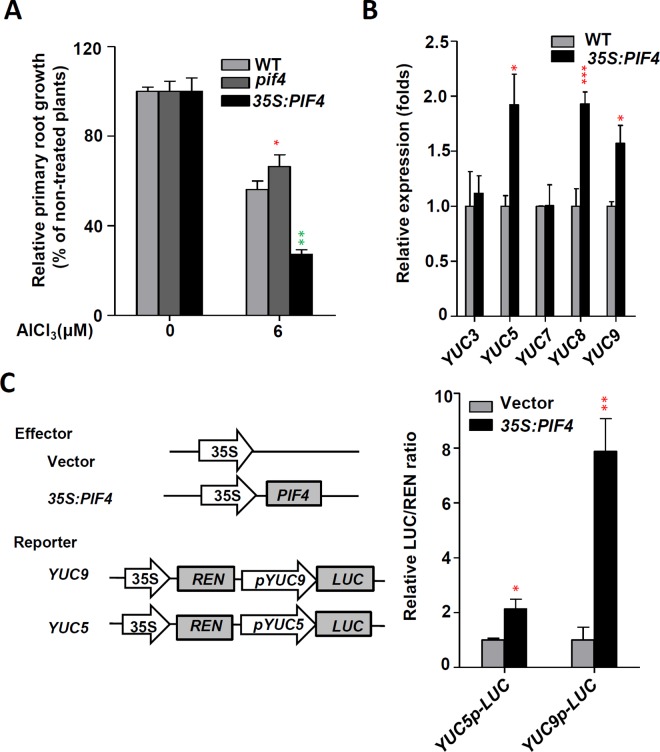
PIF4 regulates expression of *YUC*s in Al stress conditions. **(A)** Root growth of WT, *pif4* and *35S*:*PIF4* plants after a seven-day exposure to 0 or 6 μM AlCl_3_. Three independent experiments were done, each with three replicates. Plants were grown at 22°C in long days growth conditions. Error bars represent Student’s *t* test confidence intervals (n≥30). Statistical difference from expected indicated by asterisks (Fisher’s exact test, *P<0.05, **P<0.01). **(B)** Relative transcript abundance of *YUC3*, *YUC5*, *YUC7*, *YUC8* and *YUC9* genes in 7-d-old WT and *35S*:*PIF4* seedlings roots. Data represent the mean of three biological replicates. Statistical difference is indicated by asterisks (Fisher’s exact test, *P < 0.05, ***P < 0.005). **(C)** Left panel: Schematic diagrams of effector and reporter constructs used in the transient dual-luciferase assays. *CaMV 35S* promoter driving *PIF4* (*35S*:*PIF4*) was used as effector, and empty vector as a negative control. Promoter fragments of *YUC5* and *YUC9* were used to make the *YUC5p-LUC* and *YUC9p-LUC* reporters. Right panel: Transient dual-luciferase assay shows that *PIF4* transactivates the promoters of *YUC5* and *YUC9* in *Arabidopsis* protoplasts. Data represent the mean of three biological replicates. Statistical difference are indicated by asterisks (Fisher’s exact test, *P < 0.05, **P < 0.01).

Previous studies demonstrated a link between PHYTOCHROME-INTERACTING FACTOR 4 (PIF4) and auxin signalling by showing that PIF4 directly regulates the auxin biosynthetic gene *TAA1* and *YUC8* [27,28,29]. To assess the potential role of PIF4 protein in Al stress, we examined the phenotypes of the overexpression (35S:*PIF4*) and knockout (*pif4-101*) mutants of *PIF4* under Al stress treatment. We found that the root growth inhibition in the presence of Al stress was also significantly alleviated in *pif4-101* as compared with the WT, while *35S*:*PIF4* exhibited stronger inhibition of root growth in the presence of Al than the WT plants (Fig 8A). To further confirm whether PIF4 promotes Al-inhibited primary root growth via activation of *YUC* genes expression, we examined *YUC3*, *YUC5*, *YUC7*, *YUC8* and *YUC9* expression in *35S*:*PIF4* transgenic seedlings roots. Compared to the WT control, transcript levels of *YUC5*, *YUC8* and *YUC9* were elevated while the expression levels of *YUC3* and *YUC7* were not altered in the *35S*:*PIF4* seedlings roots (Fig 8B). Since PIF4 regulated *YUC8* expression has been demonstrated [28,29], here we only investigated the potential regulation of *YUC5* and *YUC9* by PIF4. Using transient expression assay in *Arabidopsis* leaves, we analysed the activation effect of PIF4 on the expression of a reporter containing the *YUC5* or *YUC9* promoters fused with the *LUC* gene. Co-expression of *YUC5p-LUC* or *YUC9p-LUC* with the *35S*:*PIF4* construct led to an obvious induction in luminescence intensity (Fig 8C), suggesting that ectopic expression of *PIF4* can activate *YUC5p-LUC* and *YUC9p-LUC* expression in this transient expression assay. Together, our assays confirmed that PIF4 not only activated *YUC8* expression [28,29], but also activated *YUC5* and *YUC9* expression. In summary, our data suggest that PIF4 is involved into Al-inhibited primary root growth by regulating *YUC5*, *YUC8* and *YUC9* expression.

### PIF4 regulates Al-induced up-regulation of YUCs and local auxin response in the root-apex TZ

**Fig 9:**
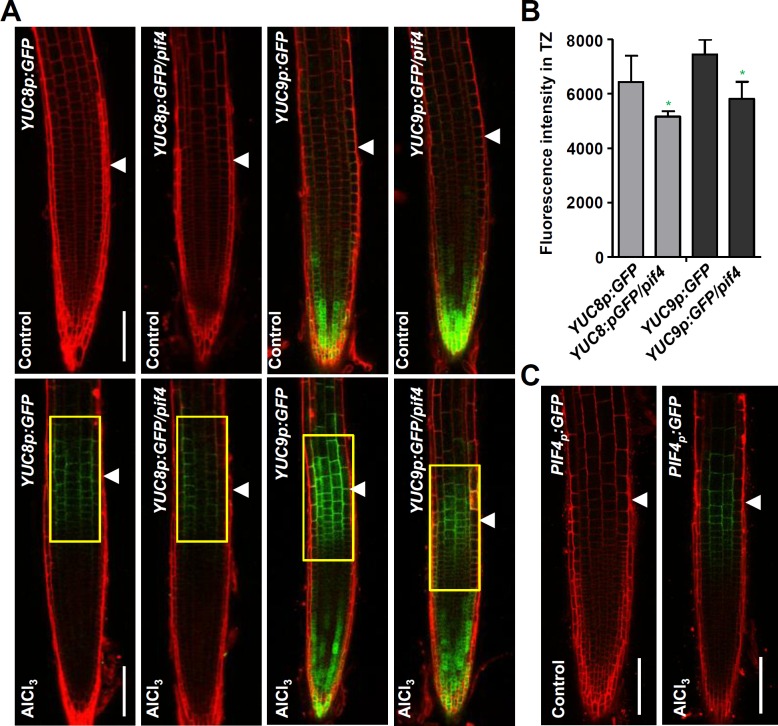
Al regulated local induction of *YUCs* in root-apex TZ is regulated by PIF4. **(A)** Five-day old *YUC8p*:*eGFP-GUS*, *YUC8p*:*eGFP-GUS/pif4-101*, *YUC9p*:*eGFP-GUS*, *YUC9p*:*eGFP-GUS/pif4101* transgenes were exposed or not (control) to 25 μM AlCl_3_ for two hours. Cell boundaries appear red following propidium iodide staining. The TZ is marked by white arrowheads. Scale bar: 100μm. **(B)** Quantification of the Al-induced fluorescence intensity in the TZ of *YUC8p*:*eGFP-GUS*, *YUC8p*:*eGFP-GUS/pif4-101*, *YUC9p*:*eGFP-GUS*, *YUC9p*:*eGFP-GUS/pif4-101* seedlings. The detected fluorescence region in TZ is marked by yellow rectangles. Cell boundaries appear red following propidium iodide staining. Error bars indicate mean ±SD (n≥20). Statistical difference from detected fluorescence is indicated by asterisks (Fisher’s exact test, *P<0.05). **(C)** The expression of *PIF4p*:*GFP* transgenes in the root-apex epidermis in presence of 25 μM AlCl_3_ for 2 hours. Cell boundaries appear red following propidium iodide staining. The TZ is marked by white arrowheads. Scale bar: 100μm.

We showed that *YUC*s are locally induced in the root-apex TZ in presence of Al stress (Fig 3 and S3 Fig). Here, PIF4 was found to be involved into root growth inhibition under Al stress. To explore the potential regulation of *YUC*s by PIF4 in this process, we analysed *YUC8p*:*eGFP-GUS* and *YUC9p*:*eGFP-GUS* expression in root-apex TZ in *pif4-101* upon Al stress. Compared with WT, the *pif4*-101 mutant displayed reduced *YUC8p*:*eGFP-GUS* and *YUC9p*:*eGFP-GUS* expression in root-apex TZ upon Al stress (Fig 9A and 9B), supporting a role of PIF4 in Al stress–induced local up-regulation of *YUCs* in root-apex TZ. This result is also consistent with the local up-regulation of *PIF4* which was shown by the increased PIF4p:GFP signals in root-apex TZ after a 2 hours-exposure to Al stress (Fig 9C).

**Fig 10:**
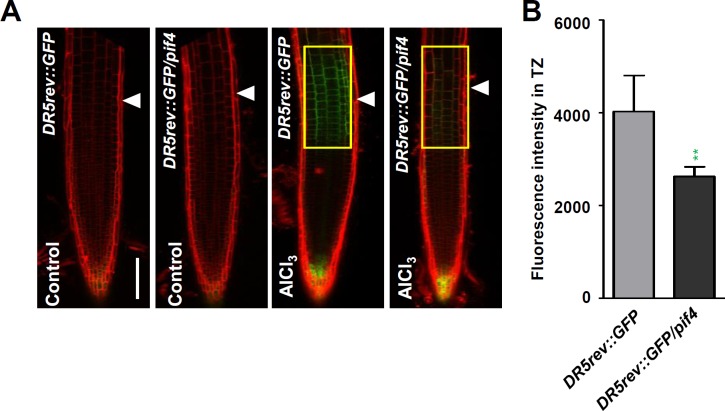
PIF4 regulates Al-induced local auxin signaling in root-apex TZ. **(A)** Five-day old *DR5rev*:*GFP* and *DR5rev*:*GFP/pif4-101* transgenes were exposed or not (control) to 25 μM AlCl_3_ for 2hours. Cell boundaries appear red following propidium iodide staining. The root-apex TZ is marked by white arrowheads. Scale bar: 100μm. **(B)** Quantification of the Al-induced fluorescence intensity in the root-apex TZ of *DR5rev*:*GFP* and *DR5rev*:*GFP/pif4-101* seedlings. The detected fluorescence region in TZ is marked by yellow rectangles. Cell boundaries appear red following propidium iodide staining. The TZ is marked by white arrowheads. Error bars indicate mean ±SD (n≥25). Statistical difference from detected fluorescence is indicated by asterisks (Fisher’s exact test, **P < 0.01).

To further test whether PIF4 affects Al-induced auxin response in the root TZ, we examined the expression of auxin responsive reporter *DR5rev*:*GFP* in the *pif4-101* mutant roots (Fig 10). The results showed that the strong Al stress-induced DR5rev:GFP signals produced in the root-apex TZ of WT root was remarkably attenuated in the *pif4-101* mutant (Fig 10). This result indicates that the reduced sensitivity of *pif4-101* root growth inhibition to the Al stress is due to an reduced auxin response in the root-apex TZ under Al stress.

### The Al-induced local expression of the *PIF4* acts downstream of ethylene signalling

**Fig 11:**
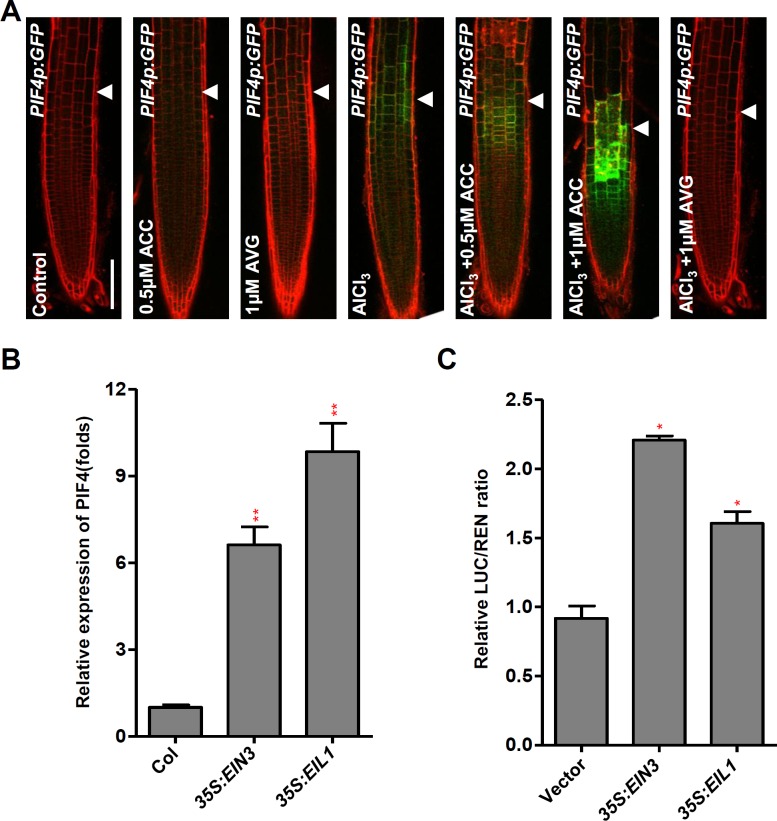
The Al-induced local expression of *PIF4* acts downstream of ethylene signaling. **(A)** The expression of *PIF4p*:*GFP* transgenes in the root apex epidermis in presence of Al and either ACC or/and AVG. Four-day old transgenic *PIF4p*:*GFP* seedlings were pre-treated with 1 μM AVG or 0.5 μM ACC for 1 day, then the seedlings were treated with 1 μM AVG, 0.5 or 1 μM ACC in the presence or not of 25 μM AlCl_3_ for 2 hours. Cell boundaries appear red following propidium iodide staining. The TZ is marked by white arrowheads. Scale bar: 100μm. **(B)** Relative transcript abundance of *PIF4* in 7-d-old Col, *35S*:*EIN3* and *35S*:*EIL1* seedlings roots. **(C)** Transient dual-luciferase assay shows that EIN3 or EIL1 transactivates the promoter of PIF4 in *Arabidopsis* protoplasts. In **(B)** and **(C)**, data represent the means of three biological replicates. Error bars represent Student’s *t* test confidence intervals (n = 3). Statistical significant difference indicated by asterisks (Fisher’s exact test, *P < 0.05, **P<0.01).

To address if ethylene signaling is involved in Al-induced up-regulation of *PIF4* in root TZ, we examined the PIF4p:GFP signals with the co-treatment of Al and ACC or AVG. The results showed that Al stress-induced up-regulation of *PIF4p*:*GFP* in the root-apex TZ was intensified by the ACC co-treatment, but repressed by the AVG co-treatment (Fig 11A). However, the supply of ACC or AVG without Al stress treatment had no effect on the expression of *PIF4* in the root tips (Fig 11A). This result suggests that the Al-induced local expression of *PIF4* is regulated by ethylene signaling. We also examined *PIF4* expression in *35S*:*EIN3* or *35S*:*EIL1* transgenic seedlings roots. Compared to the WT control, the transcript levels of *PIF4* were elevated in the *35S*:*EIN3* or *35S*:*EIL1* seedlings roots (Fig 11B). EIN3 or EIL1 can activate *PIF4* expression which was shown by the transient expression assay (Fig 11C). Though PIF4 has been shown to regulate both ethylene biosynthesis and signalling in leaf senescence [40,41], ethylene signalling may also involve in the regulation of *PIF4* expression by EIN3/EIL1 in the root under Al stress. Together, our assays demonstrated that the up-regulation of *PIF4* in the root-apex TZ under Al stress is regulated by ethylene signalling.

## Discussion

**Fig 12:**
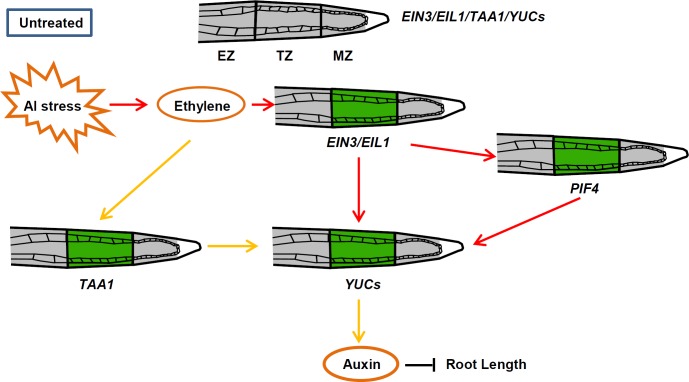
A Working model of YUC regulated root growth inhibition in response to Al stress. Al stress induces the local up-regulation of *TAA1* and *YUC*s in the root TZ through an ethylene-dependent pathway. PIF4, which is specifically up-regulated in root TZ in response to Al stress, also participates into the local up-regulation of *YUC*s. The Al–induced expression of *PIF4* in the root TZ acts downstream of ethylene signaling. The locally induced *TAA1* and *YUC*s in response to Al stress contribute to auxin accumulation in the root TZ, which suppresses primary root growth. Green represents GFP signaling; EZ: Elongation zone; TZ: Transition zone; MZ: Meristem zone.

Although the role of auxin in root growth and development was well established [42], its contribution to the response to Al stress has not yet been clarified. Auxin has been demonstrated to mediate many adaptive growth responses [43,44], and we recently have shown that exposure of the *Arabidopsis* root to Al stress induced a local up-regulation of auxin biosynthesis and thus a burst of auxin signalling in the root-apex TZ and root growth inhibition in response to Al stress. The process is dependent on TAA1, a tryptophan aminotransferase which converts Trp to IPyA, the abundance of which in the root-apex TZ rises in roots challenged by Al stress [7]. Here we demonstrate that members of the YUC family of flavin-containing mono-oxygenases, which catalyse the conversion of IPyA to IAA, a rate-limiting step in the tryptophan-dependent auxin synthesis pathway [17,20,21,45], are induced in the root-apex TZ in response to Al stress (Fig 3). Al stress-induced root growth inhibition of *yuc* mutants was strongly reduced compare with the WT control (Fig 1). Though the *yucQ* mutant was reported to have very short roots [35], both *yucQ* and WT have similar root length in MGRL solution without sucrose, which was used in this study. In addition, the *yucQ* mutant seedlings, which were grown on MS medium without sucrose, also had normal root length (S2A Fig). However, the supplementation of 1% or 5% sucrose severely inhibited root growth of the *yucQ* mutant seedlings as compared with the WT control (S2A Fig). Consistently, the highly reduced auxin response, which was shown by decreased DR5rev:GFP signals, in the root tips of *yucQ* mutant [35], was also alleviated in seedlings which were grown on MS medium without sucrose as compared with seedlings grown on MS medium supplemented with 1% or 5% sucrose (S2B Fig). The increased auxin response in the *yucQ* mutant in the absence of sucrose might be result from the up-regulation of Trp-independent auxin biosynthesis genes, which may be suppressed by sucrose. This study indicates that Al stress induces local up-regulation of YUCs and thus auxin accumulation in the root-apex TZ and root-growth inhibition (Fig 12). The implication is that plants can exploit various steps within the auxin synthetic pathway to generate the extra auxin required to reprogram the expression of the genes involved in the response to Al stress. This study also shows a good example about how environmental cues control root growth plasticity through the regulation of different steps of auxin biosynthesis and thus influencing local auxin accumulations in root-apex TZ.

In addition to auxin, ethylene, another plant hormone, also plays an important role in controlling root growth and development through cross-talk with auxin [46,47,48,49,50]. The role of ethylene in Al-stress induced root-growth inhibition has been reported [30,31]. Ethylene was found to act as a negative regulator of Al-induced malate efflux by targeting TaALMT1 (ALUMINIUM-ACTIVATED MALATE TRANSPORTER 1)-mediated malate efflux [51]. However, the exact mechanism of ethylene involvement in Al-induced inhibition of root growth is still not understood very well. And the potential participation of auxin-ethylene crosstalk in regulation of the Al-induced root growth inhibition has been suggested [31,51]. In previous reports, auxin biosynthetic genes encoding enzymes, such as WEI2/ANTHRANILATE SYNTHASE α1 (ASA1), WEI7/ASB1, TAA1, and TAR1, are regulated by ethylene [18,52], and ethylene signaling component EIN3 was suggested to directly regulate the *ASA1* based on the data of EIN3 ChIP-Seq experiments [53]. Our recent study showed that, in response to Al stress, ethylene induces local *TAA1* up-regulation and then auxin accumulation in the root-apex TZ, thus causing root growth inhibition [7]. Here, we observed that Al stress-induced local *YUCs* up-regulation in the root-apex TZ is an ethylene-dependent process (Fig 4). EIN3 acts as a regulator of Al-induced root growth inhibition [7], and Al stress induces a local expression of *EIN3/EIL1* in root-apex TZ (S7 Fig). In this study, EIN3 was found to bind to specific regions of the *YUC9* promoter (Fig 5) and Al-induced local expression of *YUCs* in the root-apex TZ is remarkably attenuated in the *ein3-1 eil1-1* mutant (S8 Fig). Al stress-induced and ethylene-dependent local up-regulation of *YUCs* contributes to auxin accumulation in the root-apex TZ and to the control of root growth inhibition (Figs 6 and 7). This study together with the previous reports suggests that auxin acts downstream and mediates ethylene regulated root-growth inhibition in response Al stress (Fig 12). It greatly improves our understanding about how ethylene mediates environmental cues and regulates plant growth adaptation through the crosstalk with auxin under stressful conditions.

PIF4, a member of the phytochrome-interacting factor (PIF) family of bHLH proteins, affects auxin-mediated growth by directly controlling the expression of *TAA1* and *YUC* genes [27,28,29]. In this study, we uncover a role for PIF4 in Al-induced root growth inhibition (Fig 8A). The increased expression of *PIF4p*:*GFP* was located predominantly in the root-apex TZ under Al stress, consistent with its role in Al-induced local up-regulation of *YUCs* and auxin accumulations in root-apex TZ (Fig 9A). Further time-course analysis of GFP signals in the presence of Al revealed that PIF4p:GFP and YUC8p:GFP signals in the TZ appeared as early as 0.5 hours (S9 Fig), suggesting that YUC may involve in an early Al-responsive signal to regulate the Al-induced root growth inhibition. Thus, as a molecular integrator, the PIF4 transcription factor links Al stress to the auxin pathway in regulating Al-induced root-growth inhibition (Fig 12). Though PIF4 has been shown to regulate both ethylene biosynthesis and signalling in the leaf senescence [40,41], ethylene signalling was shown to involve in the regulation of PIF4 expression through EIN3/EIL1 in the root under Al stress (Figs 11 and 12). The Al stress-regulated expression of *PIF4* in the root-apex TZ is modulated by an ethylene-dependent pathway (Figs 11 and 12).

TAA1 mediated local auxin biosynthesis contributes to auxin accumulation in root TZ and regulates root growth under Al stress [7]. In this study, YUCs, which act downstream of TAA1 in auxin biosynthetic pathway [20], were also found to involve in the auxin accumulation in root TZ and controls root growth under Al stress. This process was regulated by EIN3/EIL1 and PIF4 in an ethylene signalling dependent manner. Since it is well known that both auxin biosynthesis and polar auxin transport [16,54] control auxin gradient formation and regulate many plant growth and development [55,56,57]. And initial studies have been shown that polar auxin transport were also important for Al-induced root growth [31,58]. Therefore, it will be interesting to study in the future how polar auxin transport contributes to auxin gradient formation in root TZ and thus regulates root growth in response to Al stress.

## Materials and Methods

### Plant material and growth conditions

The genetic stocks used in this study are *Arabidopsis* ecotype Col-0 (WT in the text), mutants *yuc3* (GABI_376G12), *yuc7* (SALK_059832), *yuc8* (SALK_096110), *yuc9* (SAIL_871G01),*yuc9/DR5rev*:*GFP*, *yuc8 yuc9/DR5rev*:*GFP*, the transgenes *YUC5p*:*eGFP-GUS*, *YUC7p*:*eGFP-GUS*, *YUC8p*:*eGFP-GUS*, *YUC9p*:*eGFP-GUS* [24], *DR5rev*:*GFP* [59], *DR5rev*:*GFP/yuc9*, *DR5rev*:*GFP/yuc8 yuc9* [24], *DR5rev*:*GFP /yucQ* [35], *ein3-1 eil1-1* [36], *pif4-101* and *35S-PIF4* [29]. The materials were grown on Murashige and Skoog (MS) medium[60] or hydroponically grown, as described by [61], in 2% strength modified MGRL solution (pH 5.0), without inorganic phosphate and with the concentration of calcium adjusted to 200 μM as previously described [7]. This pH was chosen to maintain the Al^3+^ in solution. The plants were held at 22°C under a 16 h photoperiod.

### Treatments and analysis of root growth

*Arabidopsis* (WT and mutant) seeds were germinated for seven days on polypropylene mesh floating over the modified MGRL solution supplemented with various concentrations (0 to 6 μM) of AlCl_3_, with or without 5–(4–chlorophenyl)-4H-1,2,4–triazole-3–thiol (WAKO, 352–12001), 1-aminocyclopropane-1-carboxylic acid (ACC) (SIGMA, A3903) or aminoethoxyvinylglycine (AVG) (SIGMA, A6685) as previously described [7]. The solution was renewed every two days. Although care was taken to maintain the pH of the medium at 5.0 by regular monitoring, it was unavoidable that it dropped below this level between monitoring time points; this may explain some of the experiment-to-experiment variation in Al-induced root growth inhibition. At the end of the period, the roots were scanned and their length measured from digitized images using Image J software.

### Confocal microscopy analysis

Confocal micrographs were captured using a LSM-700 device (Zeiss, Germany). To visualize the stress-induced expression of *DR5rev*:*GFP*, *DR5rev*:*GFP/yuc9*, *DR5rev*:*GFP/yuc8 yuc9*, *DR5rev*:*GFP/yucQ*, *YUCp*:*eGFP-GUS*, *PIF4p*:*GFP*, *EIN3p*:*GFP and EIL1p*:*GFP* transgenes, seedlings were grown in non-supplemented nutrient solution for five days, then transferred to 25 μM AlCl_3_ for 2 hours as previously described [7]. The roots were stained in propidium iodide to distinguish between living and dead cells. Roots were imaged in water supplemented with propidium iodide (PI, 10 mg/L). Propidium iodide and green fluorescent protein (GFP) were viewed at excitation wavelengths of 488 nm and 561 nm, respectively. Fluorescence emission was collected at 575 nm for propidium iodide and between 500 and 530 nm band pass for GFP. The confocal microscopy assays were detected at least 30 seedlings in each experiment with or without treatments.

### Histochemical GUS staining

Staining of seedling roots for GUS activity was carried out by incubation at 37°C in 0.05M NaPO_4_ buffer (pH 7.0), 5mM K_3_Fe(CN)_6_, 5mM K_4_ Fe(CN)_6_ and 2mM X-glucuronide. Once the color had developed, the material was passed through an ethanol series (70%, 50% and 20%) before mounting in 70% chloral hydrate in 10% v/v glycerol.

### Yeast one-hybrid (Y1H) assays

Yeast one-hybrid (Y1H) assays were carried out by using the Matchmaker Gold Yeast One-Hybrid Library Screening System (Clontech). To prepare constructs for the yeast one-hybrid assay, the promoter region of *YUC9* (2 kb upstream of ATG) was amplified by PCR and cloned into the *pAbAi* vector. To generate AD-EIN3 the coding sequence of EIN3 was amplified by PCR with the respective primers and cloned into the pGAD-T7 vector (Clontech). The yeast one-hybrid assay was performed according to the Yeast Protocols Handbook (Clontech). Briefly, the bait vector was linearized and introduced into yeast strain Y1HGold to make a bait-reporter strain, then prey vector transferred into the aforementioned bait-reporter yeast strain. Transformants were grown on SD/-Leu dropout plates containing 50 or 100 ng ml^-1^ Aureobasidin A. Primers used for generating various clones are listed in S1 Table.

### ChIP-qPCR

The seedlings of 7-days-old *35S*:*EIN3-GFP* and *35S*:*GFP* [62] plants were harvested and cross-linked with 1% formaldehyde. ChIP was carried out using the EpiQuik Plant ChIP Kit (Epigentek, Brooklyn, NY, USA) with the antibody against GFP (ab290; Abcam). Input samples and immunoprecipitated samples were analyzed by qPCR. The primer sequences are listed in S1 Table. ChIP-qPCR results were first normalized with input sample. Relative enrichment was then calculated by the ratio of normalized results from *35S*:*EIN3-GFP* plants and the *35S*:*GFP* control.

### Gene expression analysis

For qRT-PCR analysis, 7-days-old seedling roots were harvested and frozen in liquid nitrogen for RNA extraction. Total RNA was extracted using RNeasy Mini Kit (Qiagen). Two microgram of total RNA was used to synthesize cDNA using Transcriptional First Stand cDNA Synthesis Kit (Roche). PCR amplification was performed with FastStart Universal SYBR Green Master (Roche) on a CFX Connect Real-Time PCR Detection System according to manufacturer's instruction (Bio-Rad). *ACTIN2* (AT3G18780) was used as an internal reference. The primers used for qRT-PCR are listed in S1 Table. The expression of each transcript was normalized against the amount of *ACTIN2* control transcript in each sample.

### Dual-luciferase transient expression assay in *Arabidopsis* protoplasts

For dual luciferase assays, promoter fragments of *YUC5*, *YUC9* and PIF4 were amplified by using specific primers and cloned into the *pGreen0800-LUC* vector [63]. The EIN3, EIL1 and PIF4 effector constructs were the *35S*:*EIN3*, *35S*:*EIL1* and *35S*:*PIF4*. For these constructs, the EIN3, EIL1 and PIF4 coding fragments were amplified by PCR and inserted into *pDONR221* (Invitrogen). Then the fragments were cloned into the GATEWAY-compatible vector *pB7WGF2*.*0* (Plant Systems Biology, VIB, University of Gent) by LR reaction. Protoplasts were isolated from *Arabidopsis* Col-0 plants as described [64] and transformed with effector constructs together with reporter constructs by the poly (ethylene glycol)-mediated method. Firefly and Renilla luciferase activities were quantified by using a dual-luciferase assay kit (Promega, USA) and detected by use of a Centro XS³ LB 960 Microplate Luminometer (BERTHOLD TECHNOLOGIES) according to the manufacturer’s instructions.

### Construction of promoter-GFP transgenic lines

The genomic fragment upstream of the *YUC3*, *PIF4*, *EIN3* or *EIL1* translation start codon were amplified by PCR and cloned into to *pDONR221* (Invitrogen). Subsequently, the fragment was cloned into GATEWAY-compatible vector *pKGWF7*.*1* (Plant Systems Biology, VIB, University of Gent) by LR reaction. The resulted plasmid were sequenced, introduced into Agrobacterium strain GV3101 or PMP90, and transformed into Col-0 plants using the floral dip method [65]. Five independent transgenic lines were examined. Primers used for the vector construction are shown in S1 Table.

### Statistical analysis

Data sets were analyzed using Prism 6 software (GraphPad Software). Comparisons between two groups were made using Student’s *t* test. Comparisons between multiple groups were made using one-way or two-way ANOVA tests depending whether one or two different variables were considered, respectively. All values were presented as mean ± SD, values of *p* less than 0.05 were considered significant. *, **, *** denote differences significant at, respectively, *P* < 0.05, < 0.01 and < 0.001.

### Accession numbers

Sequence data from this article can be found in the Arabidopsis Genome Initiative database and the GenBank/EMBL database and under the following accession numbers: *YUC3* (AT1G04610), *YUC5* (AT5G43890), *YUC7* (AT2G33230), *YUC8* (AT4G28720), *YUC9* (AT1G04180),*TAA1*(AT1G70560), *EIN3* (AT3G20770), *EIL1*(AT2G27050), *PIF4* (AT2G43010).

## Supporting Information

S1 Fig *yuc* mutants phenotypes under Al stress. **(A, B)** Root growth of WT (Col) and *yuc* mutant plants after a seven-day exposure to 0 or 6 μM AlCl_3_. Three independent experiments were done, each with three replicates. Plants were grown at 22°C in long days. Bar = 1 cm. Error bars in **(B)** represent Student’s *t* test confidence intervals (n = 40). Statistical difference from expected indicated by asterisks (Fisher’s exact test, **P<0.01).
(TIF)

S2 Fig *yucQ* mutant seedlings have no root length phenotype at the absence of sucrose. **(A)** Root growth of WT (*DR5rev*:*GFP*) plants and *DR5rev*:*GFP/yucQ* seedlings after a seven-day exposure to 0%, 1% or 5% sucrose on Murashige and Skoog (MS) medium. Three independent experiments were done, each with three replicates. Error bars indicate mean ±SD (n = 30). Statistical significance was determined by two-way ANOVA with multiple comparison correction by Duncan's multiple range test. Different letters indicate significance groups (P < 0.05). **(B)** The expression of *DR5rev*:*GFP* and *DR5rev*:*GFP/yucQ* transgenes in the root in the presence of 0%, 1% or 3% sucrose for 3 hours. Cell boundaries appear red following propidium iodide staining. Scale bar: 100μm.
(TIF)

S3 Fig Al regulated local expression of *YUC*s in the root-apex TZ. The expression of the *YUCp*:*GFP-GUS* transgenes in cortex of the roots exposed to 25 μM AlCl_3_ for two hours (lower row). Controls are untreated roots (upper row). Cell boundaries appear following propidium iodide staining. The root TZ is marked by white arrowheads. Scale bar: 100 μm.
(TIF)

S4 Fig Expression of *YUC*s are induced by Al treatment in the root-apex TZ. Five-day old *YUC5p*:*GFP-GUS* and *YUC9p*:*GFP-GUS* transgenes were exposed or not (control) to 10 μM AlCl_3_ for 3h and 6h. The TZ is marked by white arrowheads. Scale bar: 100μm.
(TIF)

S5 Fig The pH has no effect on the expression of *YUCp*:*GFP-GUS* transgenes. The expression of the *YUCp*:*GFP-GUS* transgenes (30 seedlings were detected in each material) in epidermis of the roots exposed to a pH between 4.2 and 5.9 for three hours. Controls are pH = 5.0 roots (middle row). Cell boundaries appear red following propidium iodide staining. The TZ is marked by white arrowheads. Scale bar: 100 μm.
(TIF)

S6 Fig Al regulated local induction of YUC is regulated by ethylene. The expression of *YUCp*:*GFP-GUS* transgenes in the root apex epidermis in presence of Al and either ACC or/and AVG. Four-day old transgenic *YUCp*:*GFP-GUS* seedlings were pre-treated with 1 μM AVG or 1 μM ACC for 1 day when used in co-treatment, then the seedlings were treated with 1 μM AVG or 1 μM ACC in the presence or not of 25 μM AlCl_3_ for 2 hours. Cell boundaries appear red following propidium iodide staining. The TZ is marked by white arrowheads. Scale bar: 100μm.
(TIF)

S7 Fig Al regulated up-regulation of *EIL1* and *EIN3* in the root-apex TZ. The expression of the *EIL1p*:*GFP-GUS* and *EIN3p*:*GFP-GUS* transgenes in cortex of the roots exposed to 25 μM AlCl_3_ for 0.5, 1 or 2 hours (lower row). Controls are untreated roots. The root TZ is marked by white arrowheads. Scale bar: 100 μm.
(TIF)

S8 Fig Al regulated local induction of *YUC*s is ethylene dependent. **(A)** Five-day old *YUC3p*:*GFP-GUS*, *YUC3p*:*GFP-GUS/ein3 eil1*, *YUC8p*:*GFP-GUS*, *YUC8p*:*GFP-GUS/ein3 eil1*, *YUC9p*:*GFP-GUS*, *YUC9p*:*GFP-GUS/ein3 eil1* were exposed or not (control) to 25 μM AlCl_3_ for two hours. Cell boundaries appear red following propidium iodide staining. The TZ is marked by white arrowheads. Scale bar: 100μm. **(B)** Quantification of the Al-induced fluorescence intensity in the TZ of *YUC3p*:*GFP-GUS*, *YUC3p*:*GFP-GUS/ein3 eil1*, *YUC8p*:*GFP-GUS*, *YUC8p*:*GFP-GUS/ein3 eil1*, *YUC9p*:*GFP-GUS*, *YUC9p*:*GFP-GUS/ein3 eil1* seedlings (around 25 seedlings were measured in each material). The detected fluorescence region in TZ is marked by yellow rectangles. Cell boundaries appear red following propidium iodide staining. The TZ is marked by white arrowheads. Statistical difference from detected fluorescence is indicated by asterisks (Fisher’s exact test, ** P<0.01).
(TIF)

S9 Fig Al regulated the up-regulation of PIF4 and YUC8 in the root-apex TZ. The expression of the *PIF4p*:*GFP* and *YUC8p*:*GFP-GUS* transgenes in cortex of the roots exposed to 25 μM AlCl_3_ for 0.5, 1 or 2 hours (lower row). Controls are untreated roots. The root TZ is marked by white arrowheads. Scale bar: 100 μm.
(TIF)

S1 Table Primers used in this study. (DOCX)

S1 Material EIN3 binding sites in target promoter. (DOCX)
