# Correction: Local Transcriptional Control of YUCCA Regulates Auxin Promoted Root-Growth Inhibition in Response to Aluminium Stress in *Arabidopsis*

**DOI:** 10.1371/journal.pgen.1009964

**Published:** 2021-12-08

**Authors:** Guangchao Liu, Shan Gao, Huiyu Tian, Wenwen Wu, Hélène S. Robert, Zhaojun Ding

The images for S2–S8 Figs are incorrectly switched. The image that appears as S2 Fig should be S3 Fig, the image that appears as S3 Fig should be S4 Fig, the image that appears as S4 Fig should be S5 Fig, the image that appears as S5 Fig should be S6 Fig, the image that appears as S6 Fig should be S7 Fig, the image that appears as S7 Fig should be S8 Fig, and the image that appears as S8 Fig should be S2 Fig. The figure captions appear in the correct order.

## Supporting information

S2 Fig *yucQ* mutant seedlings have no root length phenotype at the absence of sucrose. **(A)** Root growth of WT (*DR5rev*:*GFP*) plants and *DR5rev*:*GFP/yucQ* seedlings after a seven-day exposure to 0%, 1% or 5% sucrose on Murashige and Skoog (MS) medium. Three independent experiments were done, each with three replicates. Error bars indicate mean ±SD (n = 30). Statistical significance was determined by two-way ANOVA with multiple comparison correction by Duncan’s multiple range test. Different letters indicate significance groups (P < 0.05). **(B)** The expression of *DR5rev*:*GFP* and *DR5rev*:*GFP/yucQ* transgenes in the root in the presence of 0%, 1% or 3% sucrose for 3 hours. Cell boundaries appear red following propidium iodide staining. Scale bar: 100μm.
(TIF)

S3 Fig Al regulated local expression of *YUC*s in the root-apex TZ. The expression of the *YUCp*:*GFP-GUS* transgenes in cortex of the roots exposed to 25 μM AlCl_3_ for two hours (lower row). Controls are untreated roots (upper row). Cell boundaries appear following propidium iodide staining. The root TZ is marked by white arrowheads. Scale bar: 100 μm.
(TIF)

S4 Fig Expression of *YUC*s are induced by Al treatment in the root-apex TZ. Five-day old *YUC5p*:*GFP-GUS* and *YUC9p*:*GFP-GUS* transgenes were exposed or not (control) to 10 μM AlCl_3_ for 3h and 6h. The TZ is marked by white arrowheads. Scale bar: 100μm.
(TIF)

S5 Fig The pH has no effect on the expression of *YUCp*:*GFP-GUS* transgenes. The expression of the *YUCp*:*GFP-GUS* transgenes (30 seedlings were detected in each material) in epidermis of the roots exposed to a pH between 4.2 and 5.9 for three hours. Controls are pH = 5.0 roots (middle row). Cell boundaries appear red following propidium iodide staining. The TZ is marked by white arrowheads. Scale bar: 100 μm.
(TIF)

S6 Fig Al regulated local induction of YUC is regulated by ethylene. The expression of *YUCp*:*GFP-GUS* transgenes in the root apex epidermis in presence of Al and either ACC or/and AVG. Four-day old transgenic *YUCp*:*GFP-GUS* seedlings were pre-treated with 1 μM AVG or 1 μM ACC for 1 day when used in co-treatment, then the seedlings were treated with 1 μM AVG or 1 μM ACC in the presence or not of 25 μM AlCl_3_ for 2 hours. Cell boundaries appear red following propidium iodide staining. The TZ is marked by white arrowheads. Scale bar: 100μm.
(TIF)

S7 Fig Al regulated up-regulation of *EIL1* and *EIN3* in the root-apex TZ. The expression of the *EIL1p*:*GFP-GUS* and *EIN3p*:*GFP-GUS* transgenes in cortex of the roots exposed to 25 μM AlCl_3_ for 0.5, 1 or 2 hours (lower row). Controls are untreated roots. The root TZ is marked by white arrowheads. Scale bar: 100 μm.
(TIF)

S8 Fig Al regulated local induction of *YUC*s is ethylene dependent. **(A)** Five-day old *YUC3p*:*GFP-GUS*, *YUC3p*:*GFP-GUS/ein3 eil1*, *YUC8p*:*GFP-GUS*, *YUC8p*:*GFP-GUS/ein3 eil1*, *YUC9p*:*GFP-GUS*, *YUC9p*:*GFP-GUS/ein3 eil1* were exposed or not (control) to 25 μM AlCl_3_ for two hours. Cell boundaries appear red following propidium iodide staining. The TZ is marked by white arrowheads. Scale bar: 100μm. **(B)** Quantification of the Al-induced fluorescence intensity in the TZ of *YUC3p*:*GFP-GUS*, *YUC3p*:*GFP-GUS/ein3 eil1*, *YUC8p*:*GFP-GUS*, *YUC8p*:*GFP-GUS/ein3 eil1*, *YUC9p*:*GFP-GUS*, *YUC9p*:*GFP-GUS/ein3 eil1* seedlings (around 25 seedlings were measured in each material). The detected fluorescence region in TZ is marked by yellow rectangles. Cell boundaries appear red following propidium iodide staining. The TZ is marked by white arrowheads. Statistical difference from detected fluorescence is indicated by asterisks (Fisher’s exact test, ** P<0.01).
(TIF)
