# Supplementary figures and images for: Local Transcriptional Control of YUCCA Regulates Auxin Promoted Root-Growth Inhibition in Response to Aluminium Stress in Arabidopsis
Source: PLoS Genet. 2016 Oct 7;12(10):e1006360. doi: 10.1371/journal.pgen.1006360 (PMC5065128; doi:10.1371/journal.pgen.1006360)

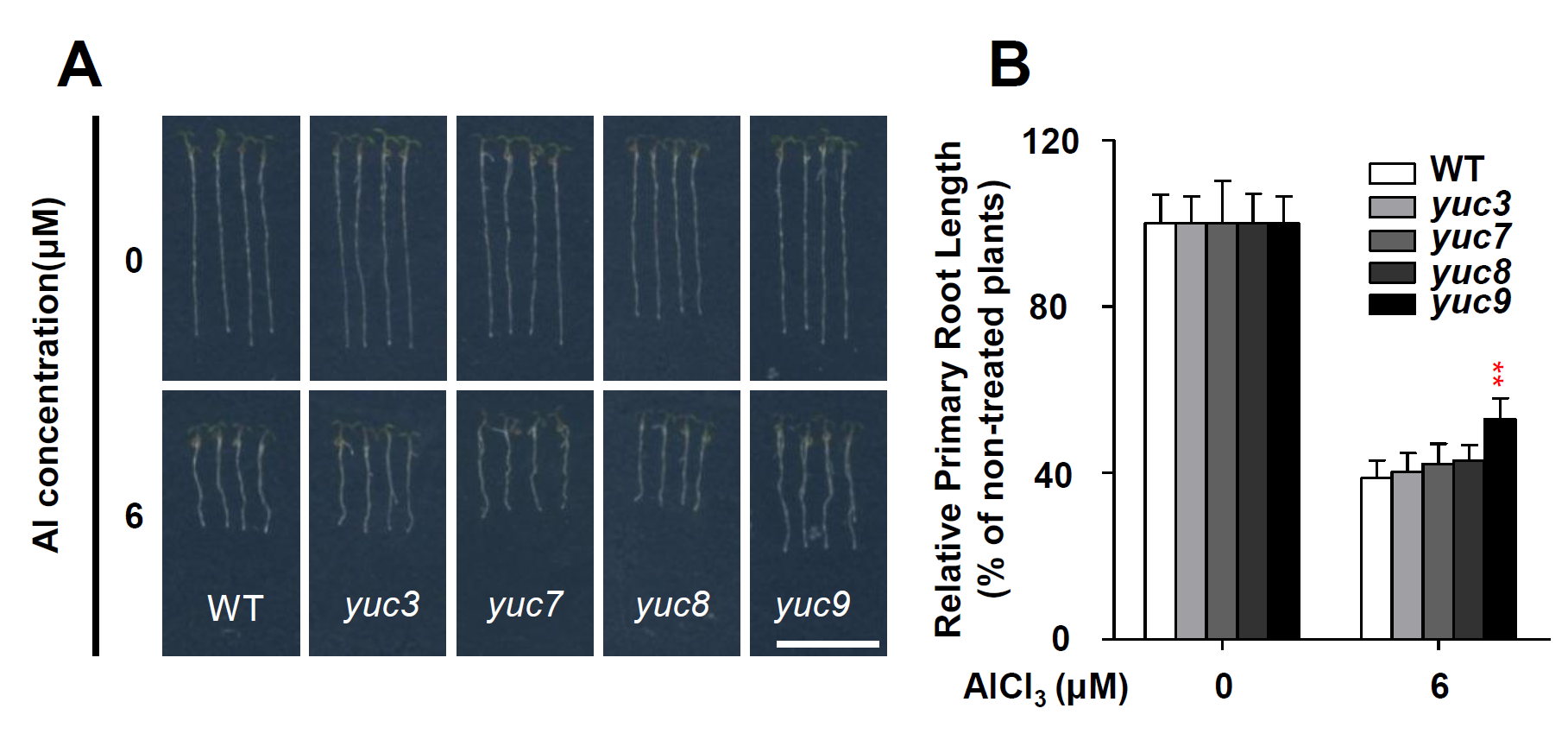

Supplement: S1 Fig — (A, B) Root growth of WT (Col) and yuc mutant plants after a seven-day exposure to 0 or 6 μM AlCl3. Three independent experiments were done, each with three replicates. Plants were grown at 22°C in long days. Bar = 1 cm. Error bars in (B) represent Student’s t test confidence intervals (n = 40). Statistical difference from expected indicated by asterisks (Fisher’s exact test, **P<0.01). (TIF) [file pgen.1006360.s001.tif]

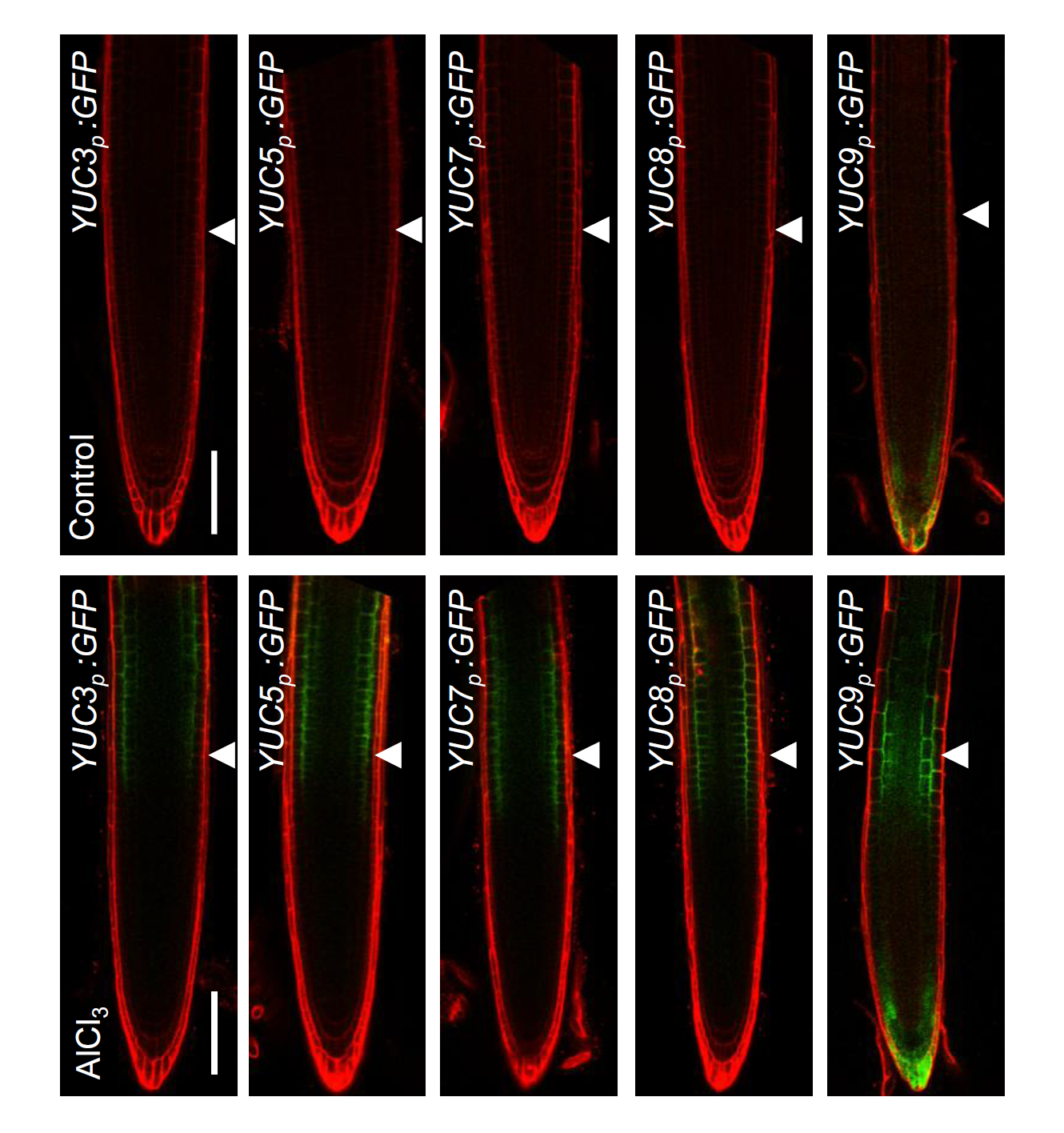

Supplement: S2 Fig — (A) Root growth of WT (DR5rev:GFP) plants and DR5rev:GFP/yucQ seedlings after a seven-day exposure to 0%, 1% or 5% sucrose on Murashige and Skoog (MS) medium. Three independent experiments were done, each with three replicates. Error bars indicate mean ±SD (n = 30). Statistical significance was determined by two-way ANOVA with multiple comparison correction by Duncan's multiple range test. Different letters indicate significance groups (P < 0.05). (B) The expression of DR5rev:GFP and DR5rev:GFP/yucQ transgenes in the root in the presence of 0%, 1% or 3% sucrose for 3 hours. Cell boundaries appear red following propidium iodide staining. Scale bar: 100μm. (TIF) [file pgen.1006360.s002.tif]

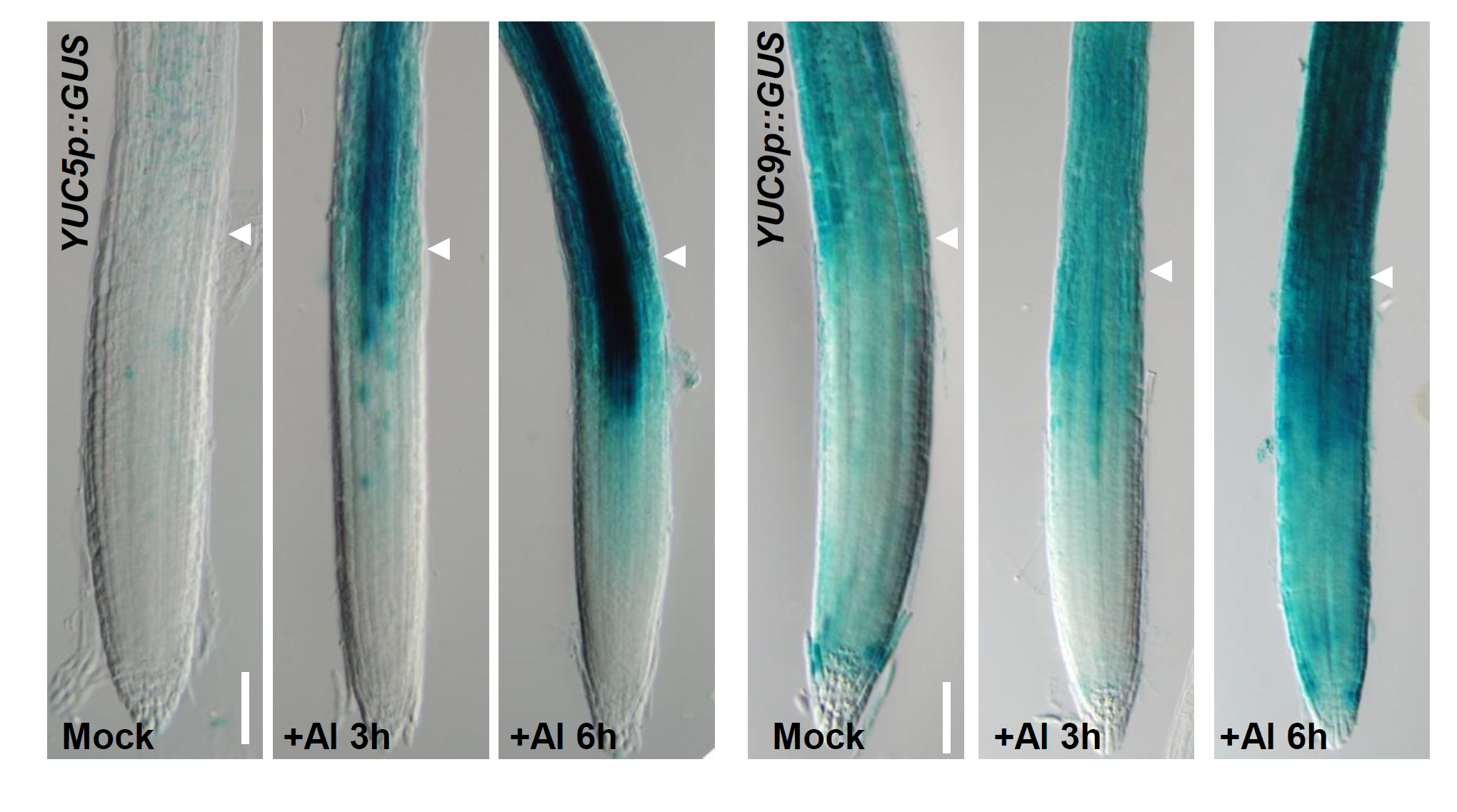

Supplement: S3 Fig — The expression of the YUCp:GFP-GUS transgenes in cortex of the roots exposed to 25 μM AlCl3 for two hours (lower row). Controls are untreated roots (upper row). Cell boundaries appear following propidium iodide staining. The root TZ is marked by white arrowheads. Scale bar: 100 μm. (TIF) [file pgen.1006360.s003.tif]

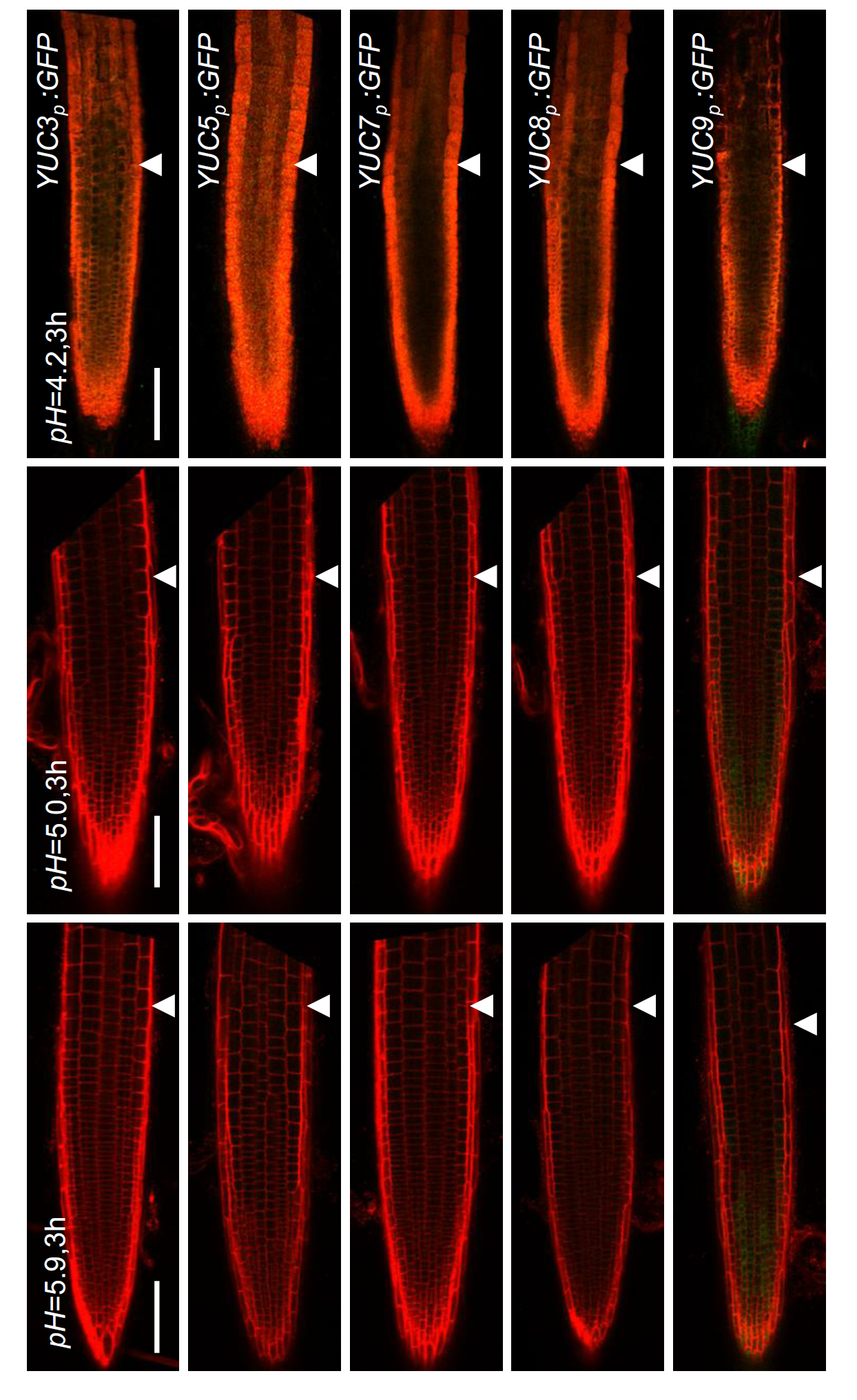

Supplement: S4 Fig — Five-day old YUC5p:GFP-GUS and YUC9p:GFP-GUS transgenes were exposed or not (control) to 10 μM AlCl3 for 3h and 6h. The TZ is marked by white arrowheads. Scale bar: 100μm. (TIF) [file pgen.1006360.s004.tif]

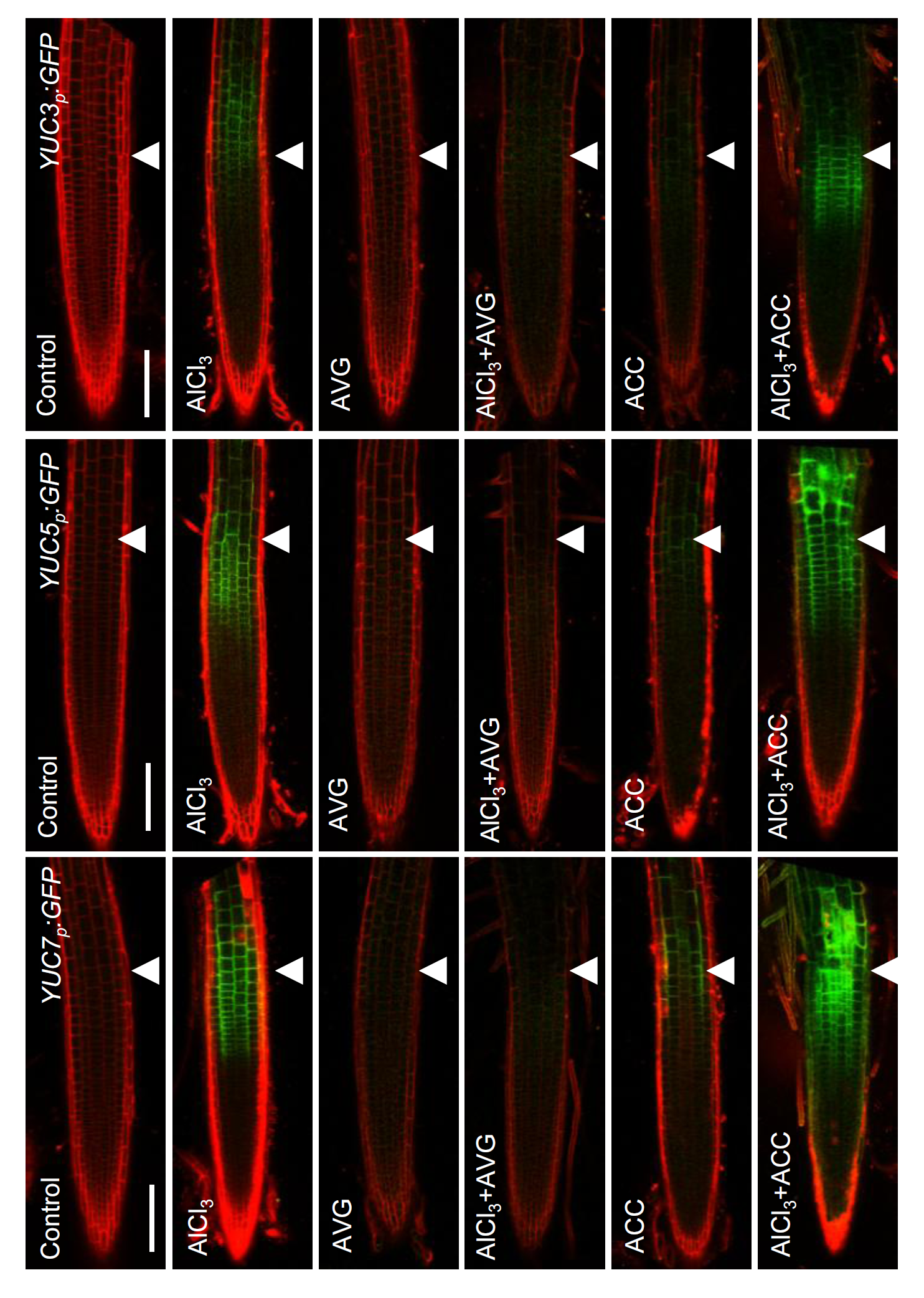

Supplement: S5 Fig — The expression of the YUCp:GFP-GUS transgenes (30 seedlings were detected in each material) in epidermis of the roots exposed to a pH between 4.2 and 5.9 for three hours. Controls are pH = 5.0 roots (middle row). Cell boundaries appear red following propidium iodide staining. The TZ is marked by white arrowheads. Scale bar: 100 μm. (TIF) [file pgen.1006360.s005.tif]

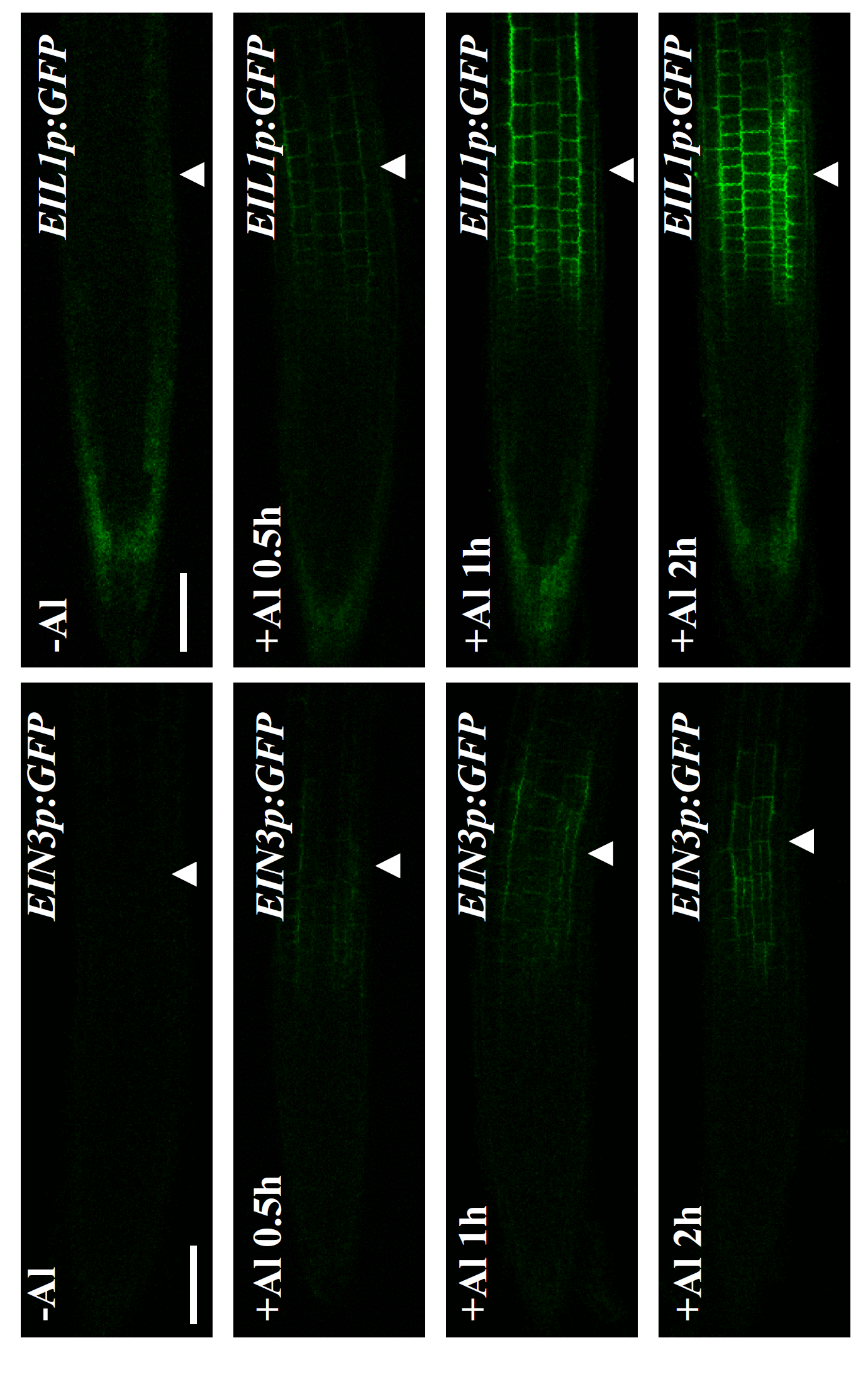

Supplement: S6 Fig — The expression of YUCp:GFP-GUS transgenes in the root apex epidermis in presence of Al and either ACC or/and AVG. Four-day old transgenic YUCp:GFP-GUS seedlings were pre-treated with 1 μM AVG or 1 μM ACC for 1 day when used in co-treatment, then the seedlings were treated with 1 μM AVG or 1 μM ACC in the presence or not of 25 μM AlCl3 for 2 hours. Cell boundaries appear red following propidium iodide staining. The TZ is marked by white arrowheads. Scale bar: 100μm. (TIF) [file pgen.1006360.s006.tif]

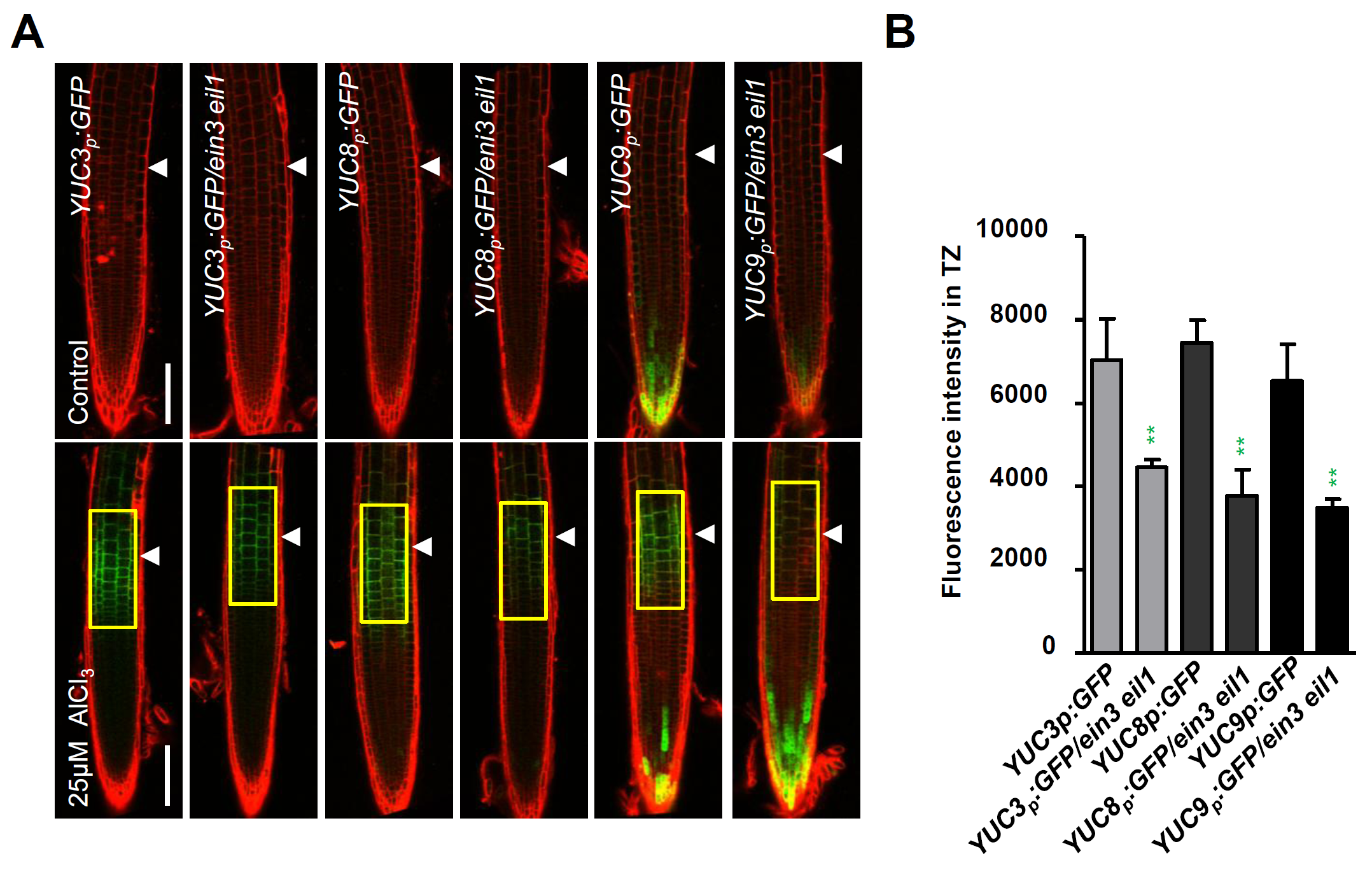

Supplement: S7 Fig — The expression of the EIL1p:GFP-GUS and EIN3p:GFP-GUS transgenes in cortex of the roots exposed to 25 μM AlCl3 for 0.5, 1 or 2 hours (lower row). Controls are untreated roots. The root TZ is marked by white arrowheads. Scale bar: 100 μm. (TIF) [file pgen.1006360.s007.tif]

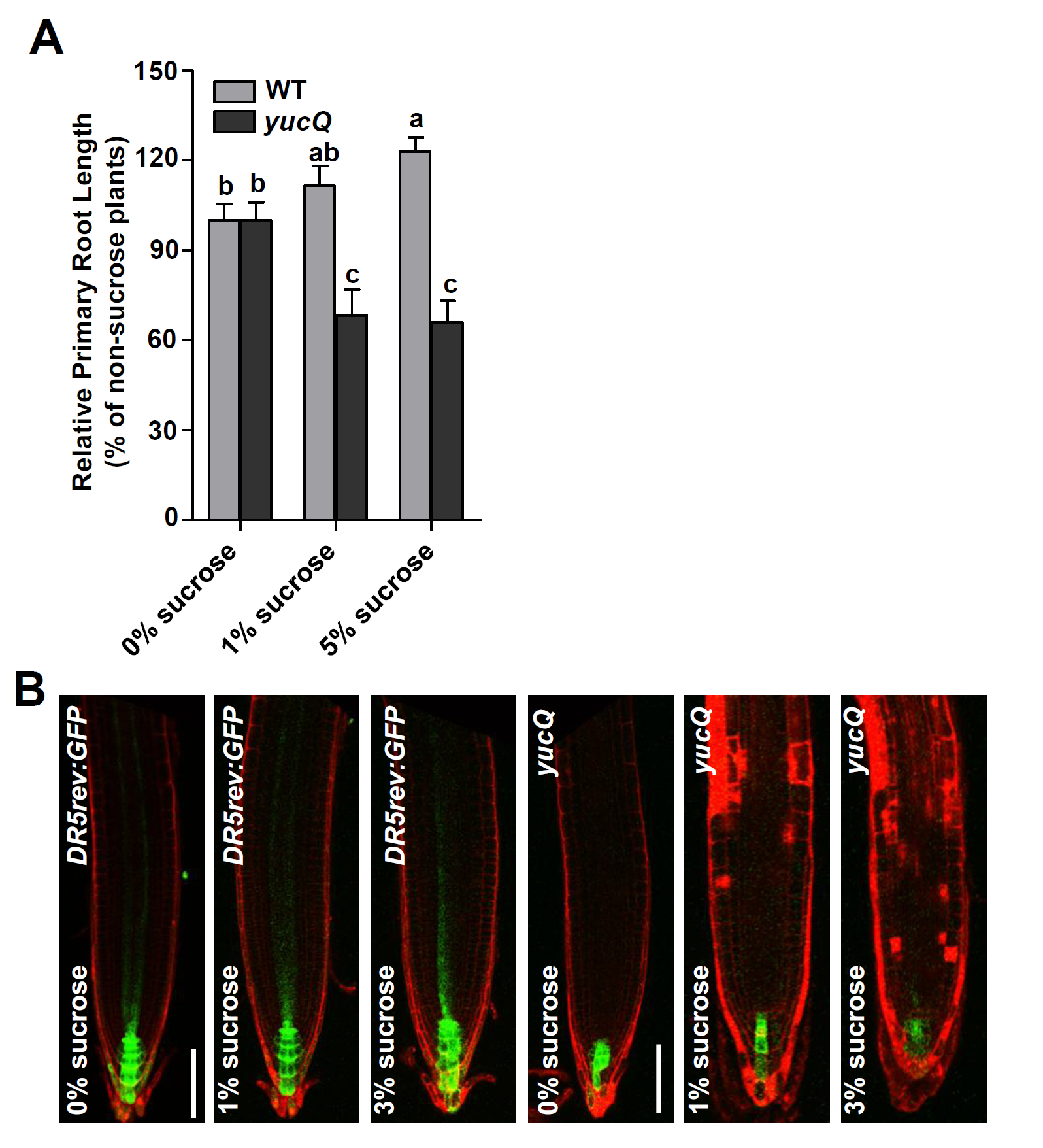

Supplement: S8 Fig — (A) Five-day old YUC3p:GFP-GUS, YUC3p:GFP-GUS/ein3 eil1, YUC8p:GFP-GUS, YUC8p:GFP-GUS/ein3 eil1, YUC9p:GFP-GUS, YUC9p:GFP-GUS/ein3 eil1 were exposed or not (control) to 25 μM AlCl3 for two hours. Cell boundaries appear red following propidium iodide staining. The TZ is marked by white arrowheads. Scale bar: 100μm. (B) Quantification of the Al-induced fluorescence intensity in the TZ of YUC3p:GFP-GUS, YUC3p:GFP-GUS/ein3 eil1, YUC8p:GFP-GUS, YUC8p:GFP-GUS/ein3 eil1, YUC9p:GFP-GUS, YUC9p:GFP-GUS/ein3 eil1 seedlings (around 25 seedlings were measured in each material). The detected fluorescence region in TZ is marked by yellow rectangles. Cell boundaries appear red following propidium iodide staining. The TZ is marked by white arrowheads. Statistical difference from detected fluorescence is indicated by asterisks (Fisher’s exact test, ** P<0.01). (TIF) [file pgen.1006360.s008.tif]

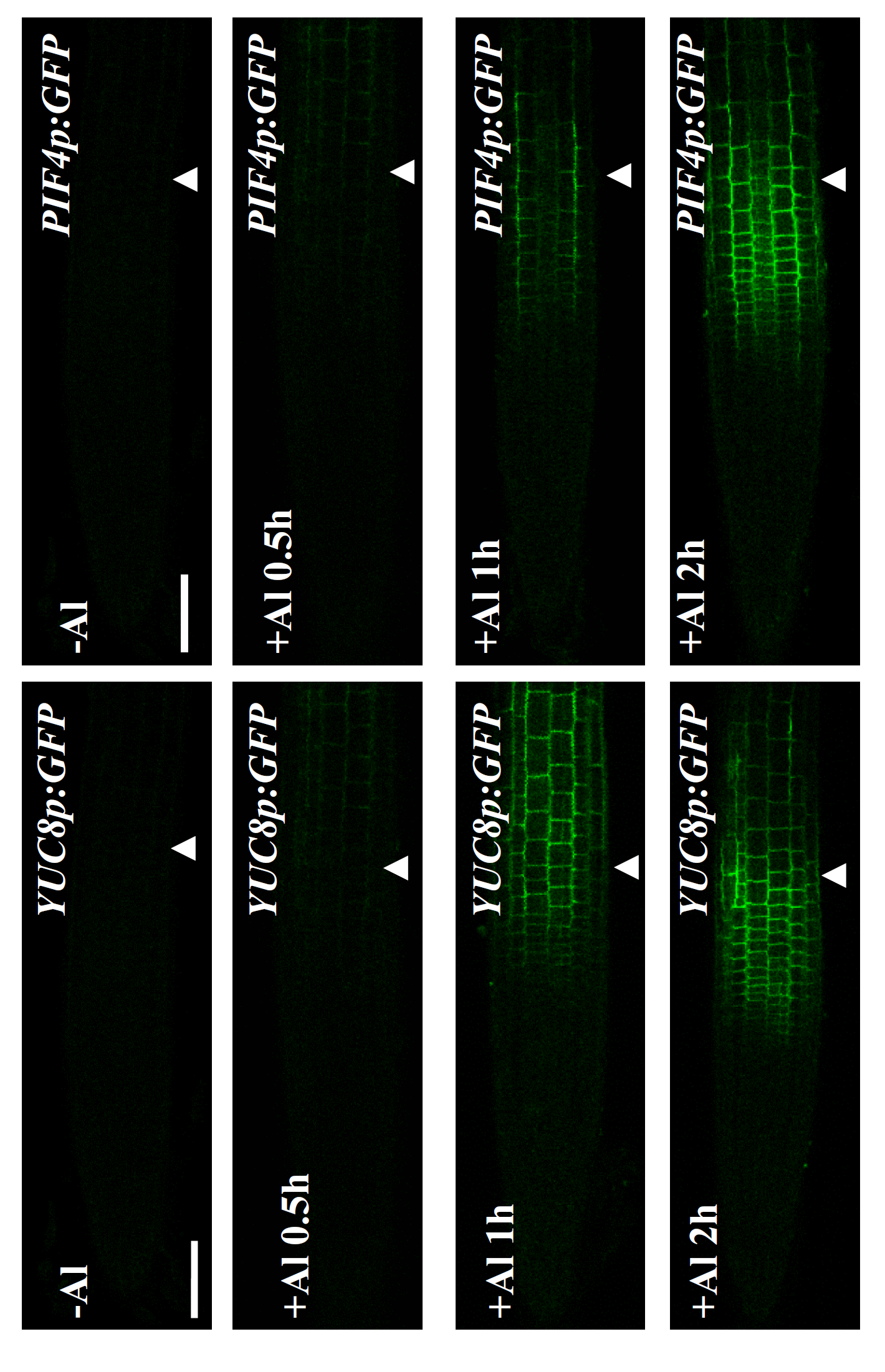

Supplement: S9 Fig — The expression of the PIF4p:GFP and YUC8p:GFP-GUS transgenes in cortex of the roots exposed to 25 μM AlCl3 for 0.5, 1 or 2 hours (lower row). Controls are untreated roots. The root TZ is marked by white arrowheads. Scale bar: 100 μm. (TIF) [file pgen.1006360.s009.tif]
