# Supplementary material for: Local Transcriptional Control of YUCCA Regulates Auxin Promoted Root-Growth Inhibition in Response to Aluminium Stress in Arabidopsis
Source: PLoS Genet. 2016 Oct 7;12(10):e1006360. doi: 10.1371/journal.pgen.1006360 (PMC5065128; doi:10.1371/journal.pgen.1006360)
Supplement: S1 Table — (DOCX) [file pgen.1006360.s010.docx]

**S1 Table. Primers used in this study**

|  | **Purpose** | **Forward Primer (5’-3’)** | **Reverse Primer (5’-3’)** |
| --- | --- | --- | --- |
| pYUC9:AbAi | Yeast assay | ATAGGTACCATACGAGGAGAAACGGGTCAG | ATACTCGAGTTTCTTGAGTGAGTTTTTGAAT |
| AD-EIN3 | Yeast assay | ATGAAGGTTAGGTTTGATCGTAATGG | TTATGAAGAATTCATAACTTTTTCTGGC |
| YUC9p:LUC | Luciferase assay | ATAGGTACCATACGAGGAGAAACGGGTCAG | ATAGGATCCTTTCTTGAGTGAGTTTTTGAAT |
| YUC5p:LUC | Luciferase assay | ATAGTCGACTACACTGCCAAACGATAG | ATAGGATCCTTTAGGGGTGAGTTTGAT |
| PIF4p:LUC | Luciferase assay | AATGGTACCAAGACAGCAAGAAAAGTAGAA | AATCTGCAGGTCAGATCTCTGGAGACA |
| 35S:EIN3 | Luciferase assay | GGGGACAAGTTTGTACAAAAAAGCAGGCTGCATGATGTTTAATGAGATGG | GGGGACCACTTTGTACAAGAAAGCTGGGTCTTAGAACCATATGGATACAT |
| 35S:EIL1 | Luciferase assay |  |  |
| 35S:PIF4 | Luciferase assay | GGGGACAAGTTTGTACAAAAAAGCAGGCTGCATGGAACACCAAGGTTGG | GGGGACCACTTTGTACAAGAAAGCTGGGTCGTGGTCCAAACGAGAACC |
| YUC3 | qRT-PCR | ATCAACCCTAAGTTCAACGAGACA | CGGCACAACTTTCTCAGCG |
| YUC5 | qRT-PCR | TGTCAATTACCCAAAATGCCC | GACCACTTGTCTCGTCGTATCG |
| YUC7 | qRT-PCR | TACCTTGAGTCCTACGCTACCC | ACCACCAAAATCTTCTAAACCCT |
| YUC8 | qRT-PCR | ATGCCCTTCCCTGAGGACTTC | TTTTCTCCCGTAGCCACCAC |
| YUC9 | qRT-PCR | ATCACTACCCTGAATACCCAACG | TTCTAACCCTCCATAGCCCG |
| PIF4 | qRT-PCR | CCAGATCATCTCCGACCGGTTTG | CTAGTGGTCCAAACGAGAACCGT |
| ACTIN2 | qRT-PCR | GGCTCCTCTTAACCCAAAGGC | CACACCATCACCAGAATCCAG |
| P1 | ChIP-qPCR | ATCTAAAAAACCTATCAGTATCACACG | TGGAGAGTGGACCAAAAGATAAT |
| P2 | ChIP-qPCR | GGGGTCCCTTTTTAGAGAGA | CATCAGGATTTGAGGTTGGTG |
| C | ChIP-qPCR | AGTCTCTCTTGATCTTGCTAACCA | TCCCCATAATCTCCCTCGG |
| YUC3:eGFP-GUS | Generate tansgenic lines | GGGGACAAGTTTGTACAAAAAAGCAGGCTTATAACTTCTCTGCCAAAGCAAGCGC | GGGGACCACTTTGTACAAGAAAGCTGGGTACTTCCACAGAGTTTAGAGTTTGCTTGTC |
| PIF4p:GFP | Generate tansgenic lines | GGGGACAAGTTTGTACAAAAAAGCAGGCTGCAAGACAGCAAGAAAAGTAGAA | GGGGACCACTTTGTACAAGAAAGCTGGGTCGTCAGATCTCTGGAGAC |
| EIN3p:GFP | Generate tansgenic lines | GGGGACAAGTTTGTACAAAAAAGCAGGCTGC CAAAACAATCAAGAAGCAT | GGGGACCACTTTGTACAAGAAAGCTGGGTCTGTAACCTGTAACAAATCAAA |
| EIL1p:GFP | Generate tansgenic lines | GGGGACAAGTTTGTACAAAAAAGCAGGCTGCTCAGGTGAGGAGGTTGG | GGGGACCACTTTGTACAAGAAAGCTGGGTCGTCTCTTCCACCACAATCAA |
