# Supplementary material for: Local Transcriptional Control of YUCCA Regulates Auxin Promoted Root-Growth Inhibition in Response to Aluminium Stress in Arabidopsis
Source: PLoS Genet. 2016 Oct 7;12(10):e1006360. doi: 10.1371/journal.pgen.1006360 (PMC5065128; doi:10.1371/journal.pgen.1006360)
Supplement: S1 Material — (DOCX) [file pgen.1006360.s011.docx]

**S1 Material.**  **EIN3 Binding Sites in Target Promoter.**

**>*YUC9* promoter**

ATCCCACTAGAACTCTATTTTACAAGAATATAATGTTCTTAGAAACCAGTTTAGAATTAACAAGTAACTTTTATTCTACTTATCTCATTTCTTAGAATGAGATTTCTAATTATTAGTAAGAACATAATTTATTTAATATATACACTGTGAGCAGCTTTTGGGTAAGAGTCATGTGAACATATATATGTCCTTGTTGCAAATTTGATCTTAACCACCACAATTATACAGCTATACTATTATATAGGTTTAATATATAATTAAATAAAATAAAACAAAGAAAGGGATAATTTTCACAAAGATCAATACAAAAGCCTTTTGGAGTATCTCCGAGGGGTCCCTTTTTAGAGAGACGATCACTGAACCTAACCAAAAAACTTAAAAATAATAATGTATCTATATACAAAGACACTATTATAGTACTCTTCTGCTTTTAAAATAAATTATGCAACACTATACATTGGCTCATATAAAATTATTGCTGACGTACCACCAACCTCAAATCCTGATGGATGAATTACACTCATAAATAACTATTATTAGCAACGTTATATATTTATAGTATTATCGATAGTTGTTGTTTACAACGGAATTCACTAGCACGATCTTTTTTTTTTTTTTTGTTAAATGGCTTTTTTCACTAGCCCGATTTACTTGCTTTTTTCTCTACAAAATAATTTTTTGTCTGTACATACAAAGCTGTGGGAGAAGAATGTATGTCGGCGTATATAGTTAGTGAAATTCTCCACTATTTCGAATTTATAATTTACGTCTACTTTTCATTTTATAACTCATATTTTACGTACGCTTGATTCCATATTCTCGAATAATCAAACAATTTTATATCATGACAACAATACATATGAACTTTACGACATGAGAATTTTATACGAGGAGAAACGGGTCAGTACGTAGAACTTGCATTACGACATACAAATATACAATATGTAAATAGCTGCAAATAATACAGCCAAATCGTGTAAATATTTATAGATATTTACTTTTTGATCTAAGTCTTTATATATAATTTCATATTCGGATGAATGAAACTCTAATTACTAAAACTAGTACTTACCTAATGGGAATACCGATTCGTGCACATGTCTATGTCATTTCCCCATGAATATTTTTTTCATCTTTGCTAATTTAAAGTTAGTGGATTACATCCATGCATGCATATATACTGTACTTCCCAAAAATATTATATGTGACGAATTTTGAGGAATTCTACGAGTTTCTAGACCTAGGTACGTTTATGATCCATTTAATACTATGAATGCTAATTTTATTAACATATCTTTTTATTAGGAACAAAAAAAGTTGTTATACAATTGCATTTAAGGAAATGAGTGCCAACTTTTATTTCATTTTTTCCAATGAAAGTCTTCAACTAAATTTAGTGTATTCCAATTTCCATGTAAGTGATATTAATATTTTTAATAAGATCCACTAAAGTATAATCAAGTATTCAAGTGGGGTAAAAGATGTAACAAACTTCCAACTATTCCCTTTGGAGATTATACAAATGTTCTCAGGGAGTGGAAATTGATATAAAACTTGAAATTGCTTAAAAGGACAAAAGAGAATGATATAGTTGCAAAAATAATAAAAAGATAGCTGCAATAATAAAATTAAGAAAGTTGCAAATTGATTTGATGTGGTTAGGTAGTCGGCGGGTGAGTAAAAAATTAAAAAGTGGGCCAACAAAATTAGGACCCGCTCTTGACTCATCATCACCTTCTCCACGTGGTTTAGTATTTGTCAGCATGACGTGCCACGTGGATTTATAAGACCAATGATTGAATTAACTCGGAGATATTAAATGATTTCTATAGGTTAGTAATTAACCTATTGGCTTATTTTTAAGTGGAGATAGTTGGCATAAACTAAAACAAACAAGTTATTCAAACTCTCTTTTATTTGGCCATAGAAAATAACAATCATATATGGTGGAGGGAATATTAGTTTGTCATAGTTTTGCCCAAAAAAAATAAAAAGTTTGTCAAATTTATTATATAAATCCAATATAAATGAGAAGAGTTTGAATAATCTGTCTTAAGAAAAAAGGTTAGTCTTAAAGTAAAAATAAAAGTAAAAGGAAACACTCTCTAGTTTCTCTACAATGGTCGTTTAGGTTCCACACTTAGAGCTTAATTTTATTTTATTTTCATACGTTTTTAACACTTTATTTTAGTTTAACCGCATTTTATGAAACTAAATTTAGCAATTTGCATGTCAGGTTGAGTTTACCATATAATTCAATCACCACGAAGAAAATAACATCTCAAATTCTAATTTCGATCAACAAATCTAAAAAACCTATCAGTATCACACGTACGTACTATTAAGACTCATCAATTTCATTCGCGTGAGTATGATCATACTGAAGCTCATGGTACTGGACCATACGATCAATTTTTTGGATGATGCGTACATGTTATGTATCTATCATATCATTATCTTTTGGTCCACTCTCCAATTTATAACCCTTTCACATCACTAAAATTCAAGTAAATTTGGTAACGAAAACCGATGGGAATCAAAATTCATACTATCAATAATCAGATTATGAAATGATAAAATAATACAGCTTATAGTTAAGGATTATTGTGAGATTTACTCTAATCAAAGTCGCACTTTGCAATAAAAGAATCGAATATTTTTATTTTTTTATACAAAAAACGTAAAGAATGTTAAATTATTTCGCATTTTAAGCAAAAGCATATGACAAAAGTTAGGTCCTAACTAAAATATTGCATACAAATTATTCACATTAATAAAATAATCAAATCCATAGAACGCGCAAATCACATGCAGTCTCGTTATAACTCTTAACCCCTATATATAGACACTCCTCTTCTCACTCCAAATCCAAACCAATAGATCTCATTTCTCATAATACAACAAAAACTAACATTCAACTTCACTACATATACTTTCAAATCGATCAAAACCCACCCTCAAAACATATACTTGCTTTCATTCAAAAACTCACTCAAGAAA

Promoter sequences were extracted from TAIR (www.arabidopsis.org/).

**EIN3 binding sites are highlighted with yellow background.**
